# Two Molecularly Defined Neuronal Types in the Mammillary Body Govern Different Temporal Periods during Working Memory Maintenance

**DOI:** 10.34133/research.1253

**Published:** 2026-04-22

**Authors:** Yiqing Guo, Lanfang Li, Zhenye Hou, Li Lu, Xiaomei Tang, Jinyu Zeng, Changdong Chai, Fuliang Jiang, Zhigao Xiang, Yuhang Shen, Aodi He, Youming Lu, Xinyan Li

**Affiliations:** ^1^ School of Basic Medicine, Tongji Medical College, Huazhong University of Science and Technology, Wuhan 430030, China.; ^2^ Innovation Center of Brain Medical Sciences, Ministry of Education of the People’s Republic of China, Wuhan 430030, China.; ^3^ Department of Plastic Surgery, Union Hospital, Tongji Medical College, Huazhong University of Science and Technology, Wuhan 430022, China.; ^4^ Department of Pathology, The Central Hospital of Wuhan, Tongji Medical College, Huazhong University of Science and Technology, Wuhan 430014, China.; ^5^ Department of Neurosurgery, Tongji Hospital of Tongji Medical College, Huazhong University of Science and Technology, Wuhan 430030, China.

## Abstract

The mammillary body (MB) has traditionally been regarded as a relay station for the hippocampus and plays a pivotal role in the Papez circuit. However, its molecular and cellular organization remains inadequately characterized. This study focuses on the horizontally symmetrically distributed neurotensin (Nts)-expressing and nitric oxide synthase 1 (Nos1)-expressing neurons in the MB, demonstrating that *Grik4* (encoding a high-affinity kainate receptor subunit) underlies their distinct electrophysiological properties. Within neural circuits, Nts and Nos1 neurons receive excitatory inputs from the ventral subiculum and send parallel excitatory projections to the dorsomedial and ventrolateral subdivisions of the anteroventral thalamus (AV). These 2 cell type-specific circuits are essential for working memory and exhibit selective activation during the maintenance phase with a marked temporal difference. Together, our findings establish a direct link from molecular identity to circuit architecture and cognitive processing by demonstrating that molecularly distinct Nts and Nos1 neurons constitute differential circuits with convergent inputs, divergent outputs, and dissociable roles in working memory maintenance. This work thus reveals a fundamental cross-scale organizational principle—molecule, cell, circuit, function—within the MB.

## Introduction

The mammillary body (MB), a compact hypothalamic nucleus located in the posterior hypothalamus [1], was first identified as a key component of the Papez circuit in 1937. This structure serves as a critical hub within limbic networks mediating memory and emotional processing [2]. Across mammalian species, the MB maintains a conserved anatomical organization, comprising 2 principal subdivisions: the larger medial mammillary nucleus (MM) and the smaller lateral mammillary nucleus (LM) [3–5]. Early investigations of MB function were significantly constrained by methodological limitations. The absence of molecularly specific neuronal markers and tracers prevented accurate cell type identification and detailed mapping of subtype-specific neural circuits, thereby impeding comprehensive understanding of MB microcircuitry.

The functional investigation of the MB traces back to 1896, when clinicopathological observations of Korsakoff’s syndrome—a condition characterized by anterograde amnesia—first identified specific organic lesions in the MB [6,7]. In 1937, neurophysiologist James Papez observed during his research on rabies that damage to the complex neural circuit composed of the hippocampus, MB, and other associated structures could lead to exaggerated emotional responses [8]. He systematically described a specific pathway interconnecting these regions, later termed the “Papez circuit” (hippocampus→MB→anterior thalamic nuclei→cingulate gyrus→parahippocampal gyrus). As a highly conserved relay hub in this circuit across species [9–11], the MB was initially thought to play a central role in emotional and cognitive regulation. Recent studies have demonstrated that the MB undergoes significant pathological changes during early Alzheimer’s disease (AD) progression, including amyloid-β accumulation, neuronal degeneration, and metabolic dysfunction [9–12]. These findings established a crucial association between MB integrity and memory processes. Subsequent research across multiple species (nonhuman primates, rats, and mice) has further confirmed the MB’s involvement in spatial navigation and episodic memory [13–16]. However, the MB’s deep anatomical location and compact size have historically constrained investigations, as conventional lesion techniques lack the precision for subregional targeting. Consequently, earlier studies primarily employed gross ablation methods, which were inadequate for resolving the functional heterogeneity of distinct MB subregions or neuronal populations in specific behavioral paradigms.

Recently, through integrated single-cell RNA sequencing and fluorescence in situ hybridization (FISH) analysis, we identified and classified 4 molecularly distinct excitatory neuronal types within the MM: parvalbumin (PV)-, dopamine receptor D2 (Drd2)-, neurotensin (Nts)-, and nitric oxide synthase 1 (Nos1)-expressing neurons [17], similar to a previous study [18]. We further demonstrated that PV and Drd2 neurons participate in distinct neural circuits and selectively mediate object and place recognition memory, respectively [17]. In contrast, the intrinsic properties of Nts and Nos1 neurons remain poorly characterized, leaving gaps in our understanding of their molecular-circuit mapping and functional roles.

In this study, we genetically targeted Nts and Nos1 neurons in the MM and found that Nos1 neurons with smaller somata were twice as abundant as their larger-soma Nts counterparts. Integrated electrophysiological and genome-wide profiling revealed that elevated expression of *Grik4*—encoding the high-affinity kainate receptor (KAR) subunit KA1—in Nos1 neurons underlies their enhanced synaptic properties, including higher miniature excitatory postsynaptic current (mEPSC) amplitude, increased α-amino-3-hydroxy-5-methyl-4-isoxazolepropionic acid receptor (AMPAR)/N-methyl-d-aspartate receptor (NMDAR) current ratio, and stronger long-term potentiation (LTP).

Neural circuit tracing revealed precisely organized connectivity patterns of Nts and Nos1 neurons. Excitatory neurons from the unilateral ventral subiculum (VS) innervated both Nts and Nos1 bilateral neuronal subgroups. In contrast, unilateral Nts and Nos1 neurons projected excitatory outputs exclusively to the ipsilateral anteroventral thalamic nucleus (AV), with distinct and parallel selectivity: Nts neurons preferentially targeted the dorsomedial AV subdivision (dmAV), while Nos1 neurons preferentially innervated the ventrolateral subdivision (vlAV).

Regarding neuronal function, we found that both Nts and Nos1 neurons—along with their associated neural circuits—play essential and incompletely overlapping roles in working memory maintenance. Using loss-of-function manipulations during a spatial working memory task [delayed nonmatch-to-place (DNMP) T maze], we demonstrated that each population contributes uniquely to this process. Due to differential *Grik4* expression, Nts neurons exhibited phase-specific activation limited to the first 20 s of working memory maintenance, whereas Nos1 neurons remained active for up to ~40 s.

## Results

### Nts and Nos1 neurons exhibit distinct spatial distribution patterns and morphological characteristics

**Fig. 1.**
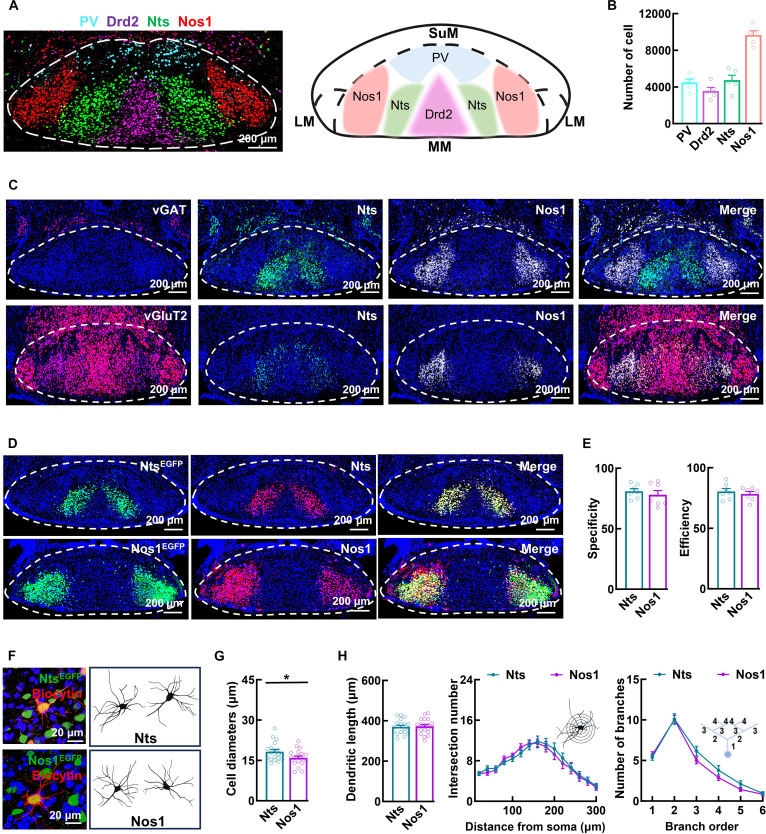
Nts and Nos1 neurons exhibit distinct spatial distribution patterns and unique morphological characteristics. (A) Representative FISH and schematic image showing the distribution patterns of PV, Drd2, Nts, and Nos1 neurons in the MM. (B) Bar graph showing the numbers of PV (4,485 ± 391), Drd2 (3,556 ± 389), Nts (4,740 ± 541), and Nos1 (9,656 ± 478) neurons. Data are mean ± SEM (*n* = 5 mice per group). (C) Representative FISH images showing Nts (green) and Nos1 (white) and vGAT (red, upper) or vGluT2 (red, bottom) in the MM. (D and E) Representative images (D) depicting the virus-labeled (VL, green) and FISH-labeled (FL, red) Nts neurons and Nos1 neurons in the MM. Bar graphs (E) showing the specificity (calculated as the proportion of FL–VL co-labeled neurons among all FL-labeled neurons) and efficiency (calculated as the proportion of FL–VL co-labeled neurons among all VL-labeled neurons) of virus labeling. Data are mean ± SEM (*n* = 7 mice per group). (F) Representative images showing EGFP-labeled and biocytin-filled (red, via patch-clamp) Nts and Nos1 neurons. (G) Graph illustrating the body size of Nts and Nos1 neurons. Data are mean ± SEM (*n* = 19 neurons from 5 mice per group; *P = 0.0360, *t* test). (H) Analysis of total dendritic length (left), dendritic complexity (middle), and branch number (right) of Nts and Nos1 neurons. Data are mean ± SEM (*n* = 19 neurons from 5 mice per group, *t* test for the bar graph and 2-way ANOVA for the line graphs).

The MM comprises 4 distinct excitatory glutamatergic neuronal types: PV-, Drd2-, Nts-, and Nos1-expressing neurons, with Nos1 neurons being approximately twice as numerous as each of the other 3 types. Unlike the dorsal–ventral distributed PV and Drd2 neurons in the medial regions of the MM, both Nts and Nos1 neurons form bilaterally symmetric subgroups distributed along the horizontal axis in the lateral regions, with Nos1 neurons positioned lateral to Nts neurons (Fig. 1A to C). To investigate the properties of Nts and Nos1 neurons, we employed a CRE-dependent genetic labeling strategy by stereotactically injecting recombinant adeno-associated virus (rAAV2/9-hSyn-DIO-EGFP) into the MM of Nts^CRE^ and Nos1^CRE^ mice, resulting in highly specific and efficient EGFP (enhanced green fluorescent protein) expression in Nts and Nos1 neurons (Fig. 1D and E). Morphological analysis showed that both neuronal types exhibited small, reticular granule cell-like features (Fig. 1F and G and Movies S1 and S2). However, Nts neurons displayed marginally larger somata (18.25 ± 0.79 μm) compared to Nos1 neurons (15.97 ± 0.69 μm), although this size difference was not accompanied by variations in total dendritic length, complexity, or branch number (Fig. 1H).

### Nts and Nos1 neurons demonstrate different electrophysiological properties

**Fig. 2.**
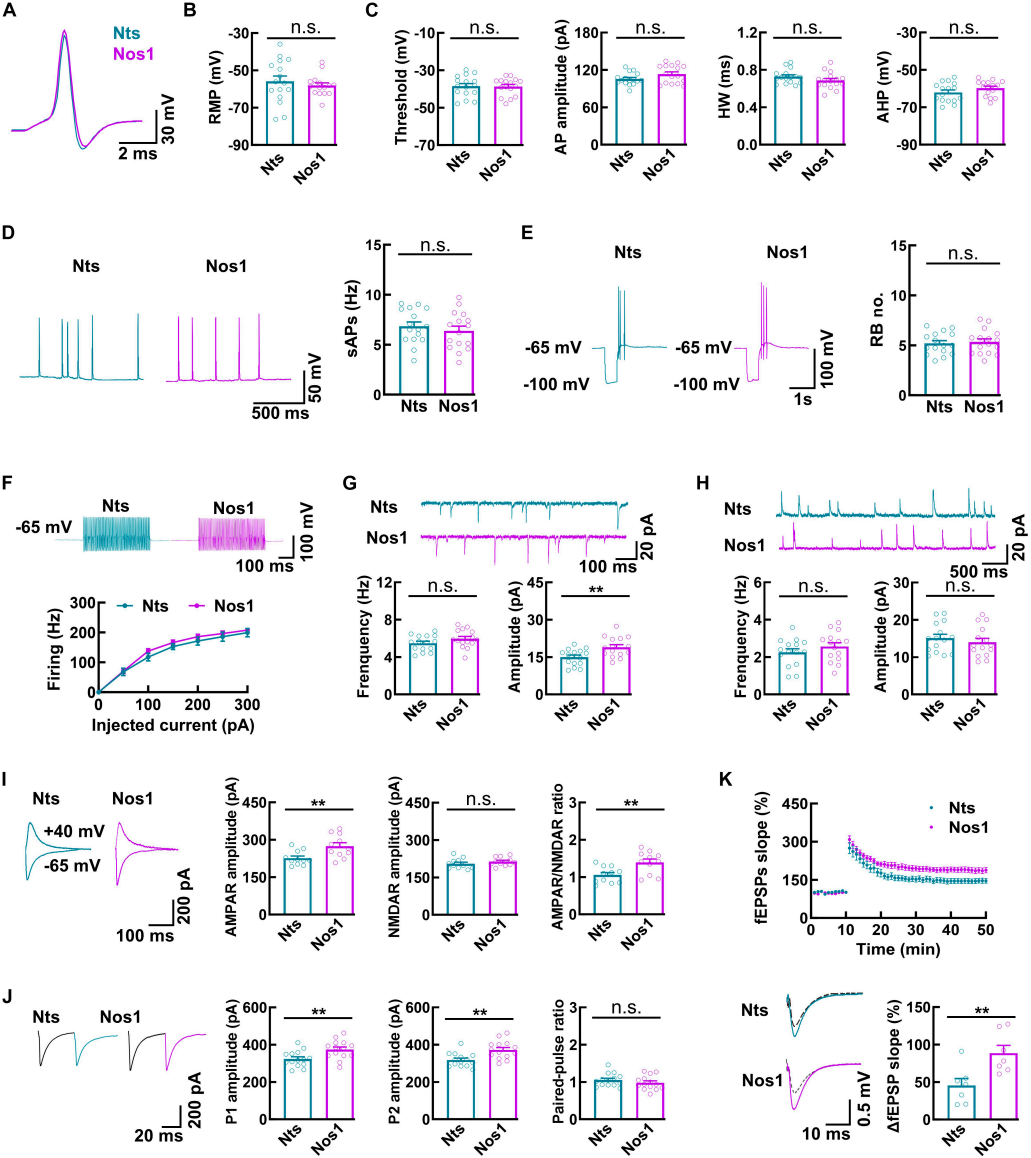
Nts and Nos1 neurons demonstrate different electrophysiological properties. (A to C) Representative traces (A) and bar graphs showing the resting membrane potential (RMP; B) and action potentials (C) recorded from Nts and Nos1 neurons in slices of the MM. Data are mean ± SEM (*n* = 16 neurons from 4 mice per group, *t* test). (D) Representative traces and bar graphs showing the spontaneous action potentials (sAPs) recorded from Nts and Nos1 neurons. Data are mean ± SEM (*n* = 16 neurons from 4 mice per group, *t* test). (E) Representative traces and bar graphs showing the rebound bursts (RB) recorded from Nts and Nos1 neurons. Data are mean ± SEM (*n* = 16 neurons from 4 mice per group, *t* test). (F) Representative traces and a plot showing the frequencies of evoked spiking activities in Nts neurons and Nos1 neurons in response to different current injections. Data are mean ± SEM (*n* = 14 neurons from 4 mice in Nts group, *n* = 17 neurons from 4 mice in Nos1 group, *t* test). (G and H) Representative traces and bar graphs showing mEPSCs (G) and mIPSCs (H) in Nts and Nos1 neurons. Data are mean ± SEM (*n* = 15 neurons from 4 mice per group; **P = 0.0056, *t* test). (I) Representative traces and bar graphs showing AMPAR- and NMDAR- mediated EPSCs recorded from Nts and Nos1 neurons. Data are mean ± SEM (*n* = 11 neurons from 4 mice per group, AMPAR amplitude: **P = 0.0078, AMPAR/NMDAR ratio: **P = 0.0072, *t* test). (J) Representative traces and bar graphs showing AMPAR-mediated EPSCs induced by paired stimulation (50-ms interval). Data are mean ± SEM (*n* = 14 neurons from 4 mice per group, P1 amplitude: **P = 0.0087, P2 amplitude: **P = 0.0028, *t* test). (K) Plots and representative traces showing the LTP characterization across Nts and Nos1 neurons. The dotted traces signify the base amplitude of field excitatory post-synaptic potentials (fEPSPs) 0 to 10 min prior to higher frequency stimulation, while the solid traces represent the amplitude of fEPSPs 40 to 50 min after higher frequency stimulation. Data are presented as mean ± SEM (*n* = 7 slices from 4 mice per group; **P = 0.0091, *t* test).

Using ex vivo electrophysiology, we recorded EGFP-labeled Nts and Nos1 neurons and found no significant differences in resting membrane potential, action potential threshold, amplitude, half-width (HW), and after hyperpolarization potential (AHP) (Fig. 2A to C). Moreover, both spontaneous and evoked spiking activities were comparable between the 2 neuronal types (Fig. 2D and E). Notably, similar to PV and Drd2 neurons in the MM, both Nts and Nos1 neurons exhibited fast-spiking properties (up to ~200 Hz), a characteristic feature typically associated with inhibitory neurons (Fig. 2F).

To investigate potential differences in synaptic function, we systematically characterized neurotransmission efficacy in Nts and Nos1 neurons. Analysis of synaptic transmission revealed that only the amplitude of mEPSCs, a sensitive indicator of postsynaptic modifications, differed between Nts and Nos1 neurons (14.93 ± 0.86 in Nts neurons versus 18.97 ± 1.04 in Nos1 neurons; Fig. 2G). Neither mEPSC frequency nor miniature inhibitory postsynaptic current (mIPSC) parameters showed significant differences (Fig. 2G and H).

Given that either postsynaptic AMPAR or NMDAR can regulate mEPSC amplitude dynamics, we performed evoked current recordings to examine potential receptor differences. Nos1 neurons demonstrated significantly larger AMPAR-mediated currents compared to Nts neurons (226.20 ± 8.6 in Nts neurons versus 274.20 ± 13.78 in Nos1 neurons), while NMDAR-mediated currents remained comparable between the 2 neuronal types. Consequently, Nos1 neurons exhibited higher AMPAR/NMDAR current ratio, despite showing similar AMPAR-mediated paired-pulse ratio (PPR) (Fig. 2I and J). Furthermore, LTP experiments revealed enhanced synaptic plasticity in Nos1 neurons relative to Nts neurons (Fig. 2K). Together, these results demonstrate distinct synaptic transmission profiles between Nts and Nos1 neurons, particularly in AMPAR-mediated postsynaptic responses and synaptic plasticity mechanisms.

### *Grik4* underlies the electrophysiological differences between Nts and Nos1 neurons

**Fig. 3.**
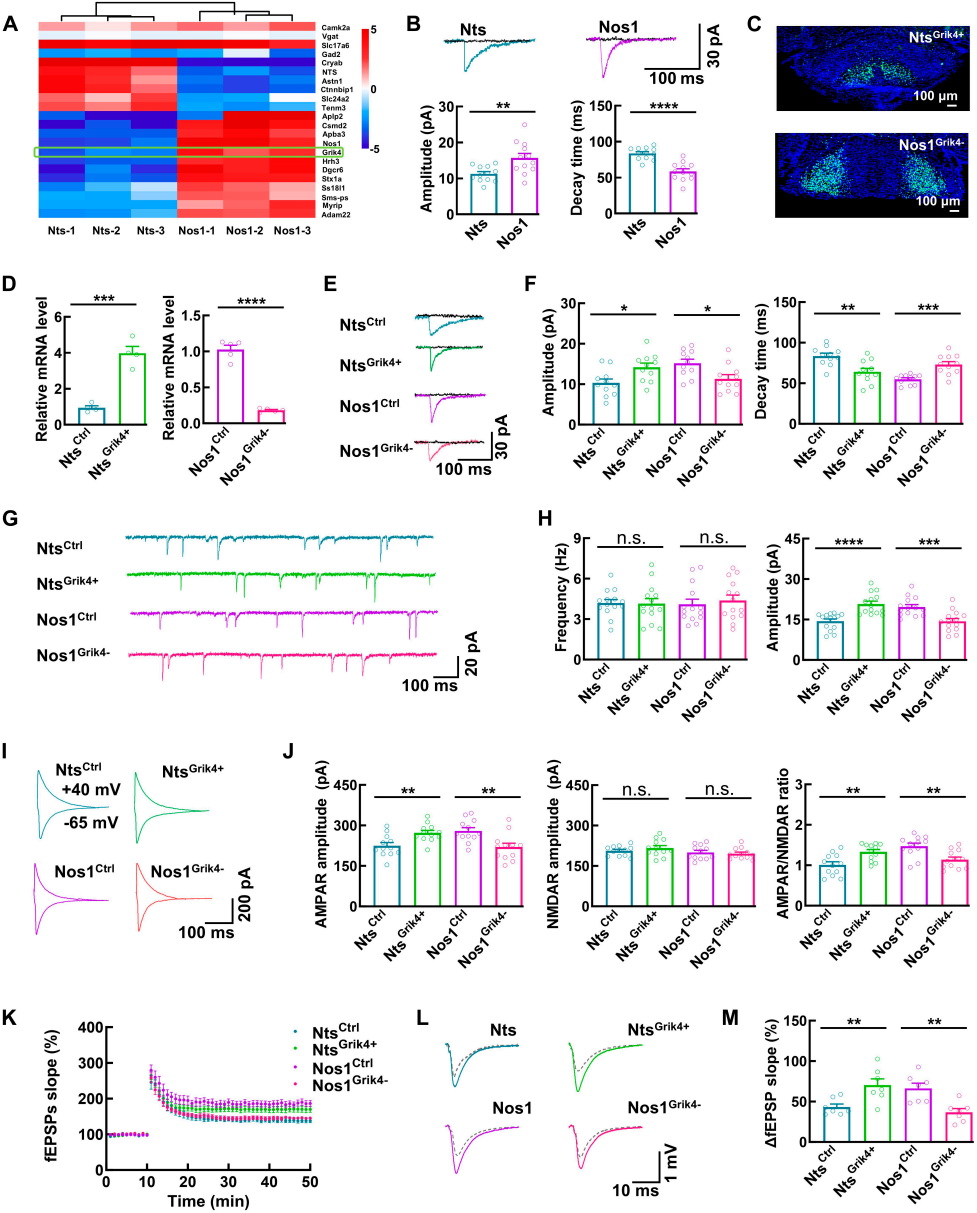
*Grik4* underlies the electrophysiological differences between Nts and Nos1 neurons. (A) Heat plot showing the genes enriched in Nts versus Nos1 neurons (*n* = 3 biological replicates per group). (B) Representative traces and bar graphs illustrating KAR-mediated EPSCs in Nts and Nos1 neurons, along with their amplitude and decay time. The currents (blue and purple traces) were blocked by the KAR/AMPA receptor antagonist CNQX (50 μM, black traces). Data are mean ± SEM (*n* = 12 neurons from 3 mice per group, Amplitude: **P = 0.0056, Decay time: ****P < 0.0001, *t* test). (C) Representative images showing *Grik4* overexpression in Nts neurons and knockdown in Nos1 neurons by respectively injecting rAAV2/9-hSyn-DIO-*Grik4*-EGFP (rAAV2/9-hSyn-DIO-EGFP as control) and rAAV2/9-hSyn-DIO-EGFP-shRNA-*Grik4* (rAAV2/9-hSyn-DIO-EGFP-shRNA-scramble as control) into the MM of Nts^CRE^ and Nos1^CRE^ mice. (D) Bar graphs showing relative *Grik4* mRNA levels using qPCR after overexpression in Nts neurons and knockdown in Nos1 neurons. Data are mean ± SEM (*n* = 4 in Nts^Ctrl^ and Nts^Grik4+^ group, *n* = 5 in Nos1^Ctrl^ and Nos1^Grik4−^ group; Nts^Ctrl^ versus Nts^Grik4+^ ***P = 0.0003, Nos1^Ctrl^ versus Nos1^Grik4−^ ****P < 0.0001, *t* test). (E and F) Representative traces (E) and bar graphs (F) illustrating KAR-mediated EPSCs in Nts^Ctrl^, Nts^Grik4+^, Nos1^Ctrl^, and Nos1^Grik4−^ neurons, along with their amplitude and decay time. Data are mean ± SEM (*n* = 11 neurons from 4 mice per group, Amplitude: Nts^Ctrl^ versus Nts^Grik4+^ *P = 0.0130, Nos1^Ctrl^ versus Nos1^Grik4−^ *P = 0.0130, Decay time: Nts^Ctrl^ versus Nts^Grik4+^ **P = 0.0018, Nos1^Ctrl^ versus Nos1^Grik4−^ ***P = 0.0002, *t* test). (G and H) Representative traces (G) and bar graphs (H) showing mEPSCs and the frequency and amplitude of mEPSCs in Nts^Ctrl^, Nts^Grik4+^, Nos1^Ctrl^, and Nos1^Grik4−^ neurons. Data are mean ± SEM (*n* = 14 neurons from 4 mice per group, Amplitude: Nts^Ctrl^ versus Nts^Grik4+^ ****P < 0.0001, Nos1^Ctrl^ versus Nos1^Grik4−^ ***P = 0.0008, *t* test). (I and J) Representative traces (I) and bar graphs (J) showing AMPAR- and NMDAR-mediated EPSCs recorded from Nts^Ctrl^, Nts^Grik4+^, Nos1^Ctrl^, and Nos1^Grik4−^ neurons. Data are mean ± SEM (*n* = 12 neurons from 4 mice per group, AMPAR amplitude: Nts^Ctrl^ versus Nts^Grik4+^ **P = 0.0045, Nos1^Ctrl^ versus Nos1^Grik4−^ **P = 0.0040, AMPAR/ NMDAR ratio: Nts^Ctrl^ versus Nts^Grik4+^ **P = 0.0033, Nos1^Ctrl^ versus Nos1^Grik4−^ **P = 0.0027, *t* test). (K to M) Plots (K and M) and representative traces (L) showing the LTP characterization across the Nts^Ctrl^, Nts^Grik4+^, Nos1^Ctrl^, and Nos1^Grik4−^ neurons. Data are mean ± SEM (*n* = 7 slices from 4 mice per group; Nts^Ctrl^ versus Nts^Grik4+^ **P = 0.0087, Nos1^Ctrl^ versus Nos1^Grik4−^ **P = 0.0028, *t* test).

We performed RNA sequencing (RNA-seq) to characterize the gene expression profiles of Nts and Nos1 neurons. The analysis confirmed selective enrichment of *Nts* and *Nos1* transcripts in their respective neuronal populations (Fig. 3A). Both neuronal types expressed canonical markers of excitatory glutamatergic neurons, including Ca^2+^/calmodulin-dependent kinase IIα (CaMKIIα) and vesicular glutamate transporter-2 (vGluT2), while showing no detectable expression of inhibitory neuronal markers (GAD1, GAD2, and vGAT) (Fig. 3A). These results were consistent with the FISH data and our published findings [17]. Comparative transcriptome analysis identified 38 and 217 significantly enriched genes in Nts and Nos1 neurons, respectively (Fig. S1A and Tables S1 and S2).

Notably, *Grik4* emerged as a prominently differentially expressed gene, showing particularly high expression in Nos1 neurons (Fig. S1B). *Grik4* encodes the KA1, a high-affinity subunit to KAR. Increased *Grik4* expression promotes the integration of KA1 subunits into KAR. These KA1-containing KARs exhibit enhanced agonist affinity and specific channel kinetics, characterized by rapid activation and decay [19,20]. Previous studies have established that KA1-containing KAR can potentiate both AMPAR-mediated synaptic responses and synaptic plasticity [19,21–23]. Quantitative polymerase chain reaction (qPCR) validation confirmed higher *Grik4* expression in Nos1 neurons compared to Nts neurons (Fig. S1C). Electrophysiological recordings further revealed that Nos1 neurons exhibited KAR-mediated currents with significantly larger amplitudes and faster decay kinetics, characteristic functional signatures of KA1-containing KAR (Fig. 3B).

To establish a causal relationship between *Grik4* expression and neuronal physiology, we performed gain- and loss-of-function experiments. *Grik4* overexpression in Nts neurons (Nts^Grik4+^) and knockdown in Nos1 neurons (Nos1^Grik4−^) were verified at both transcriptional (qPCR) and functional levels (Fig. 3C to F). Remarkably, Nts^Grik4+^ neurons acquired electrophysiological properties indistinguishable from control Nos1 neurons (Nos1^Ctrl^), demonstrating increased mEPSC amplitudes, larger evoked AMPAR-mediated currents, and enhanced synaptic plasticity. Conversely, Nos1^Grik4−^ neurons exhibited physiological characteristics comparable to control Nts neurons (Nts^Ctrl^) (Fig. 3G to M). Further analysis of the ratio of post- to pre-LTP induction currents showed that the amplitude of KAR-mediated currents remained stable after LTP induction, irrespective of *Grik4* expression (Fig. S1D and E). In contrast, the amplitude of AMPAR-mediated currents was significantly enhanced following LTP induction, with a more pronounced increase observed in *Grik4*-rich neurons (Fig. S1F and G). Consistent with prior reports that elevated *Grik4* expression enhances the proportion of calcium-permeable AMPARs [21], we conclude that the enhanced LTP is caused by *Grik4* overexpression, which leads to increased AMPAR permeability. These complementary experiments provide compelling evidence that differential *Grik4* expression is a major determinant of the distinct synaptic transmission properties distinguishing Nts and Nos1 neurons.

### Nts and Nos1 neurons form distinct neural circuits

Previous studies have demonstrated that the MB receives input from the hippocampus and projects to the anterior thalamic nucleus, but the neuron type-specific circuitry involving Nts and Nos1 neurons remains unclear. To elucidate these connections, we employed retrograde and anterograde monosynaptic tracing techniques.

**Fig. 4.**
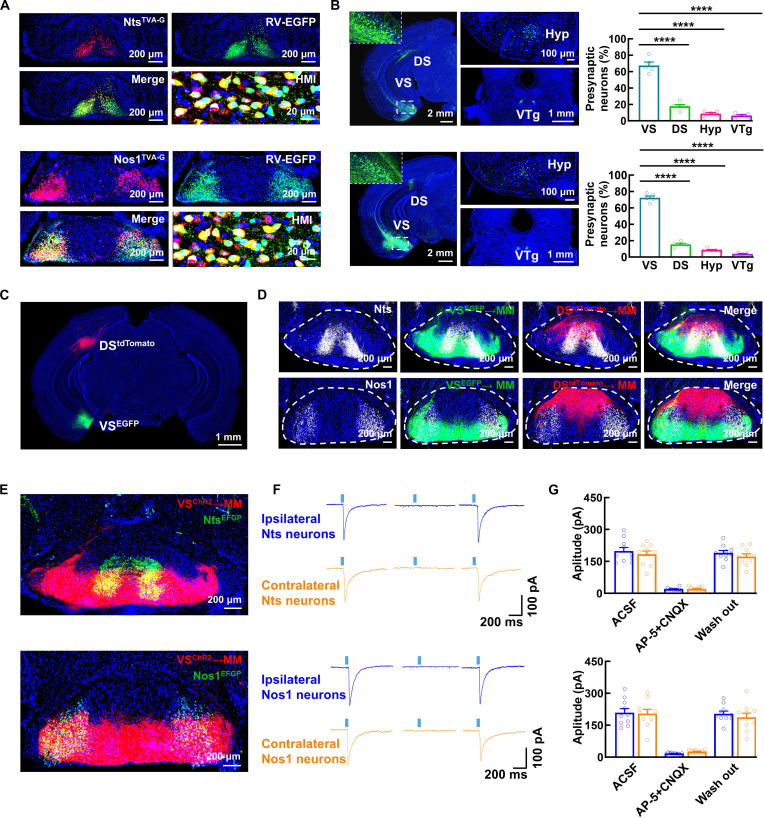
Unilateral VS innervates bilateral Nts and Nos1 neurons. (A and B) Representative images showing EGFP in Nts^TVA-G^ neurons and Nos1^TVA-G^ neurons (A) and their presynaptic neurons throughout the brain (B) by injecting rAAV2/9-hSyn-DIO-mCherry-TVA-G and EnvA-RV(CVS-N2C)-ΔG-EGFP into the MM of Nts^CRE^ and Nos1^CRE^ mice. Bar graphs (B) showing the quantitative distribution of EGFP-labeled presynaptic neurons in each of the indicated brain regions. Data are mean ± SEM (*n* = 5 mice per group, Nts: VS versus DS, VS versus Hyp, and VS versus VTg presynaptic neurons: ****P < 0.0001; Nos1: VS versus DS, VS versus Hyp, and VS versus VTg presynaptic neurons: ****P < 0.0001, one-way ANOVA). HMI, high-magnification image. (C and D) Representative images showing Nts and Nos1 neurons, and tdTomato- and EGFP-labeled neurons in the unilateral DS and VS (C) and their axons in the MM (D) by respectively injecting rAAV2/9-hsyn-tdTomato and rAAV2/9-hsyn-EGFP into DS and VS of wild-type mice. Nts and Nos1 neurons were stained using FISH. (E) Representative images showing ChR2-labeled axons from VS (red) and EGFP-labeled Nts or Nos1 neurons in the MM by respectively injecting rAAV2/9-hsyn-ChR2(H134R)-tdTomato into the unilateral VS and rAAV2/9-hSyn-DIO-EGFP into the MM of Nts^CRE^ and Nos1^CRE^ mice. (F and G) Representative traces (F) and bar graphs (G) showing the optically evoked postsynaptic currents (oEPSCs) from both unilateral and contralateral Nts and Nos1 neurons. oEPSCs were evoked by optically stimulating the unilateral VS^ChR2^ axon in the MM, and blocked by CNQX/AP-5. Data are mean ± SEM (*n* = 10 neurons from 4 mice per group).

We selectively targeted Nts and Nos1 neurons in the MM of Nts^CRE^ and Nos1^CRE^ mice by injecting rAAV2/9-hSyn-DIO-mCherry-TVA-G to express TVA and rabies glycoprotein (G). Subsequent application of the modified rabies virus (RV) (EnvA-RV-ΔG-EGFP) enabled monosynaptic retrograde tracing of presynaptic inputs (Fig. 4A). EGFP-labeled presynaptic neurons were identified in the dorsal and ventral subiculum (DS and VS), the hypothalamus (Hyp), and the ventral tegmental nuclei of Gudden’s tegmental nucleus (VTg) (Fig. 4B). Importantly, within the hippocampus, labeling was restricted to the DS and VS, with no significant signal detected in other hippocampal subregions. These brain regions also correspond to the findings of other researchers [24,25].

For both Nts and Nos1 neurons, the majority of presynaptic inputs originated from the VS, a finding corroborated by anterograde tracing showing that DS and VS axons preferentially innervate the dorsal minority and ventral majority of Nts and Nos1 neurons, respectively. Notably, unilateral VS neurons project into the MM via an ipsilateral axonal pathway, with axons distributed broadly along its horizontal axis (Fig. 4C and D and Fig. S2A). To further characterize these connections, we expressed channelrhodopsin-2 (ChR2) and tdTomato in unilateral VS neurons and EGFP in Nts and Nos1 neurons (Fig. 4E). The majority of bilateral Nts and Nos1 neurons receive direct excitatory input from unilateral VS neurons, as demonstrated by optogenetic stimulation combined with electrophysiological recordings (Fig. 4F and G and Fig. S2B and C). These currents were tetrodotoxin (TTX)-sensitive and reversible upon 4-AP application, and further confirmed by monosynaptic latency [26,27] (Fig. S2D and E). Immunohistochemical validation confirmed that presynaptic VS neurons are excitatory pyramidal neurons (Fig. S2F).

**Fig. 5.**
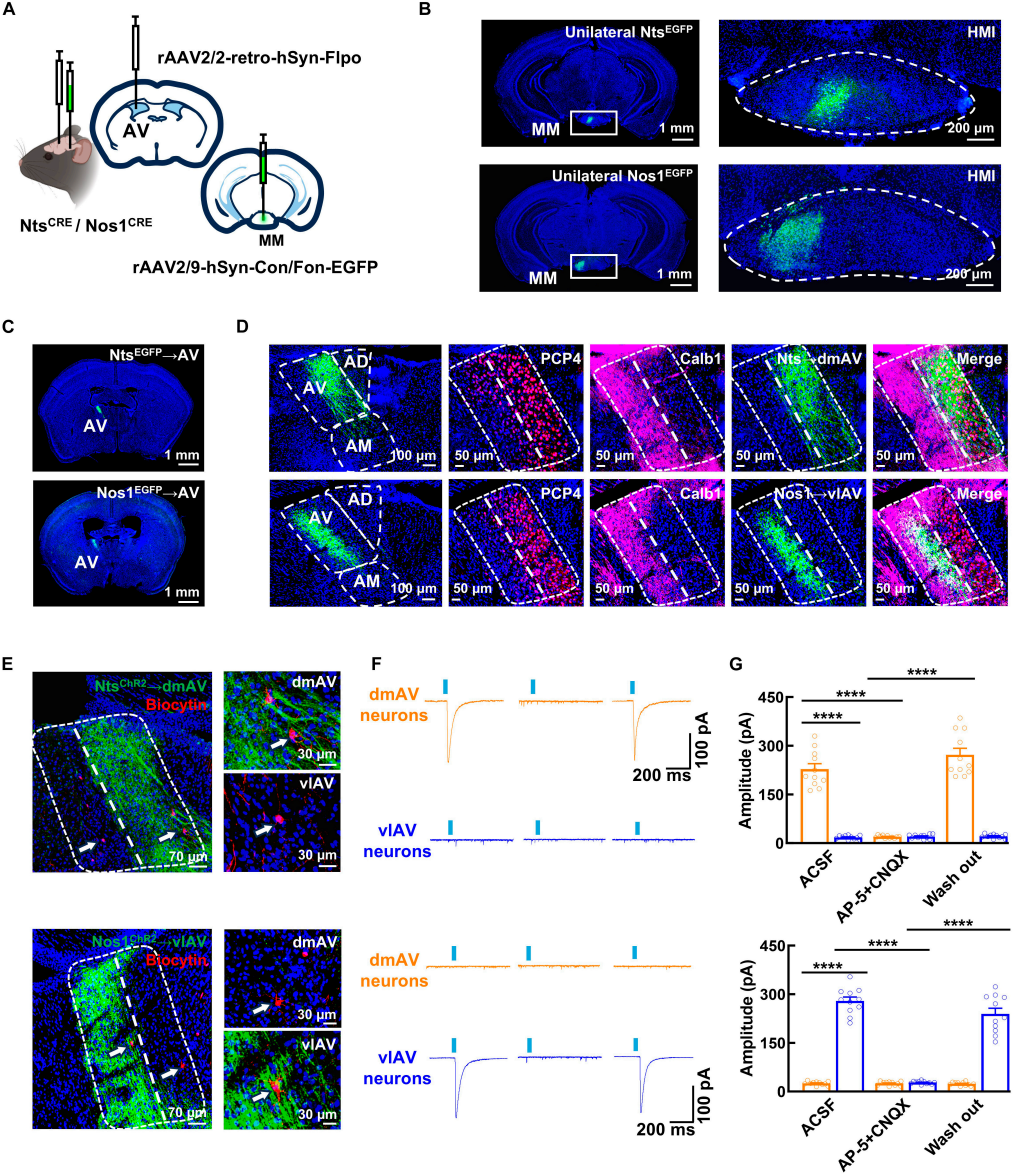
Nts and Nos1 neurons parallelly project to the ipsilateral dmAV and vlAV. (A and B) Schematic of viral strategy (A) and representative images (B) for unilaterally labeling Nts and Nos1 neurons by injecting rAAV2/2-retro-hSyn-Flpo into the unilateral AV and rAAV2/9-hSyn-Con/Fon-EGFP into the MM of Nts^CRE^ and Nos1^CRE^ mice. (C and D) Representative images showing the axons from unilaterally EGFP-labeled Nts neurons and Nos1 neurons in the AV (C) and co-staining with anti-PCP4 and anti-Calb1 (D). (E) Representative images showing ChR2-labeled axons (green) from unilateral Nts and Nos1 neurons [injection of rAAV2/2-retro-hSyn-Flpo into the unilateral AV and rAAV2/9-hSyn-Con/Fon-ChR2(H134R)-EGFP into the MM of Nts^CRE^ and Nos1^CRE^ mice] and biocytin-filled neurons (red, via patch-clamp) in the AV. (F and G) Representative traces (F) and bar graphs (G) showing the oEPSCs from dmAV and vlAV neurons by optically stimulating the Nts^ChR2^ or Nos1^ChR2^ axon in the AV. Data are mean ± SEM (*n* = 11 neurons from 4 mice per group, ACSF, ACSF versus AP-5 + CNQX, and AP-5 + CNQX versus wash out: Nts group ****P < 0.0001, Nos1 group ****P < 0.0001, one-way ANOVA).

Given the established role of the anterior thalamic nuclei as a critical node in the Papez circuit for memory processing, and its known receipt of MM projections [1,28], we focused our analysis on characterizing the differential inputs from Nts and Nos1 neurons. Anterograde tracing combined with Nts and Nos1 neuronal labeling revealed that both Nts and Nos1 neurons project to the AV (Fig. S3A). Due to the close proximity of bilateral Nts and Nos1 neuronal subgroups in the MM, conventional CRE-dependent viral injections cannot selectively target unilateral subgroups. To precisely map these projections, we first performed retrograde tracing from the unilateral AV, which showed that MM-originating presynaptic neurons were restricted to the ipsilateral side (Fig. S3B and C). Following this, a dual-virus strategy was employed to achieve unilateral labeling: injecting rAAV2/2-retro-hsyn-Flpo into the AV and rAAV2/9-hSyn-Con/Fon-EGFP into the MM of Nts^CRE^ and Nos1^CRE^ mice (Fig. 5A). This revealed that both Nts and Nos1 neurons project exclusively to the ipsilateral AV but target distinct molecularly defined subregions—Nts neurons innervate the PCP4 (Purkinje cell protein 4)-positive dorsomedial AV (dmAV), while Nos1 neurons project to the Calb1 (Calbindin1)-positive ventrolateral AV (vlAV) [29] (Fig. 5B to D). Optogenetic and electrophysiological analyses confirmed that these parallel pathways (Nts→dmAV and Nos1→vlAV) exhibit excitatory monosynaptic connectivity (Fig. 5E to G and Fig. S3D and E).

Bilateral experiments, including neural circuit tracing and optogenetics-assisted electrophysiology, yielded symmetrical connectivity patterns. Collectively, our findings delineate the precise anterograde and retrograde connectivity of Nts and Nos1 neurons: They receive unilateral-to-bilateral input from VS and send unilateral-to-ipsilateral parallel projections to the dmAV and vlAV, with all connections being monosynaptic and excitatory (Fig. S3F). Our results reveal a divergent projection pattern of Nts neurons (Nts→dmAV), contrasting with the previously reported projection to the anteromedial thalamic nucleus (AM) [18]. The robustness of our findings is established through FISH-confirmed neuronal labeling (validated against the Allen Brain Atlas), integrated with bidirectional viral tracing (anterograde/retrograde) of the Nts→dmAV projection and optogenetically validated functional connectivity, collectively confirming the precision of our neuronal circuit characterization.

Previous research has demonstrated a bidirectional connection between the VTg and MM: VTg provides inhibitory projections to the MM, while the MM sends excitatory projections back to the VTg [30]. However, the precise connectivity pattern remained unclear. Using anterograde and retrograde tracing of unilateral Nts and Nos1 neurons in the MM, we found that both Nts and Nos1 neurons project ipsilaterally to the VTg, and that unilateral VTg neurons project bilaterally to Nts and Nos1 neurons (Fig. S4).

### Nts and Nos1 neurons govern different temporal periods during working memory maintenance

**Fig. 6.**
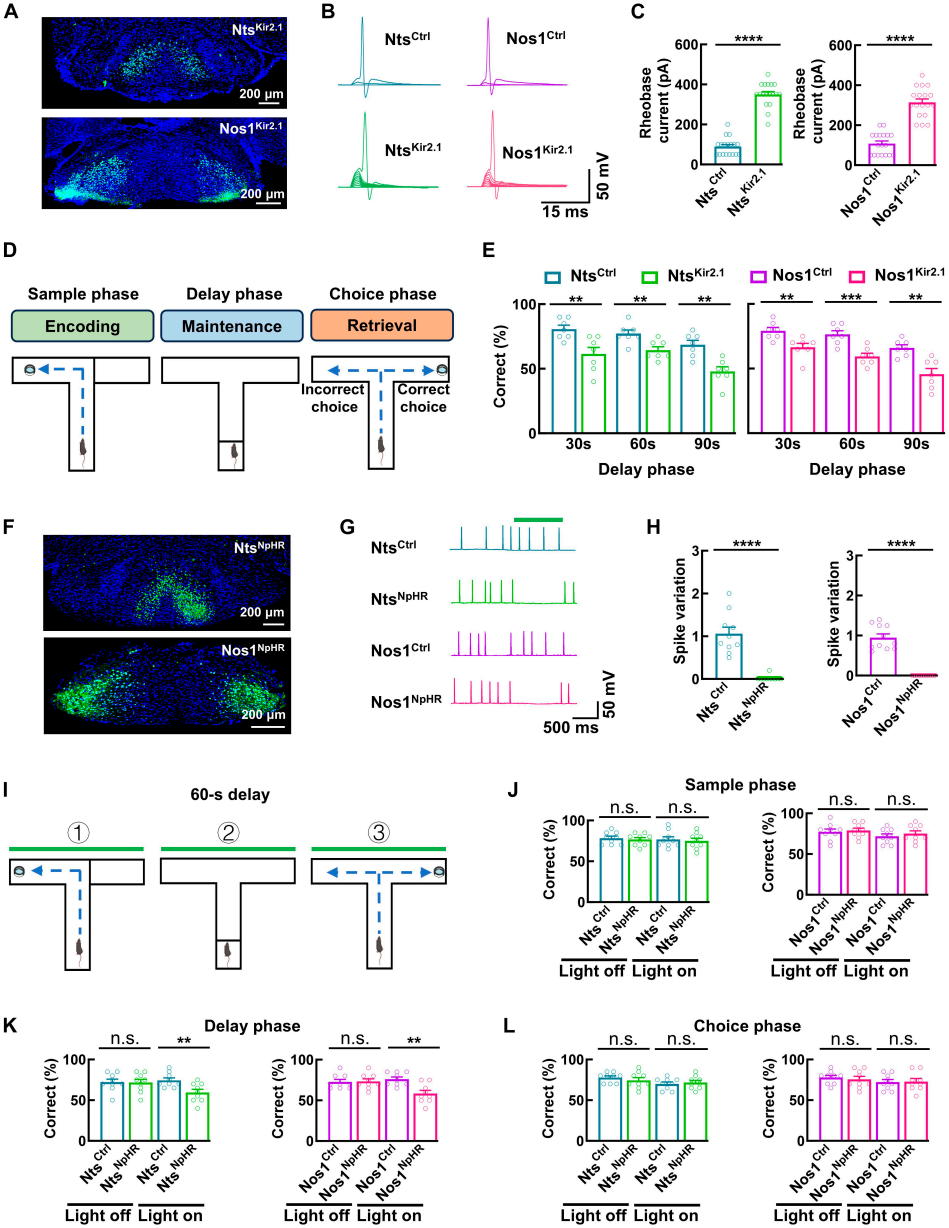
Inhibition of Nts and Nos1 neurons impairs working memory. (A) Representative images showing the expression of Kir2.1 in Nts and Nos1 neurons by injecting rAAV2/9-hSyn-DIO-Kir2.1-EGFP (rAAV2/9-hSyn-DIO-EGFP as control) into the MM of Nts^CRE^ and Nos1^CRE^ mice. (B and C) Representative traces (B) and bar graphs (C) showing rheobase current of AP recorded from the Nts^Ctrl^, Nts^Kir2.1^, Nos1^Ctrl^, and Nos1^Kir2.1^ neurons. Data are mean ± SEM (*n* = 18 neurons from 6 mice per group, Nts^Ctrl^ versus Nts^Kir2.1^ ****P < 0.0001, Nos1^Ctrl^ versus Nos1^Kir2.1^ ****P < 0.0001, *t* test). (D) Schema of the DNMP T maze and its associated memory phases. (E) Bar graphs showing the performance of Nts^Ctrl^, Nts^Kir2.1^, Nos1^Ctrl^, and Nos1^Kir2.1^ mice in the testing sessions of DNMP T maze with 30-, 60-, and 90-s delay phase. Data are mean ± SEM (*n* = 7 mice per group, Nts^Ctrl^ versus Nts^Kir2.1^: 30 s **P = 0.0067, 60 s **P = 0.0072, and 90 s **P = 0.0013; Nos1^Ctrl^ versus Nos1^Kir2.1^: 30 s **P = 0.0070, 60 s ***P = 0.0007, and 90 s **P = 0.0013, *t* test). (F) Representative images showing the expression of NpHR in Nts and Nos1 neurons by injecting rAAV2/9-hSyn-DIO-NpHR3.0-EGFP (rAAV2/9-hSyn-DIO-EGFP as control) into the MM of Nts^CRE^ and Nos1^CRE^ mice. (G and H) Representative traces (G) showing the spontaneous action potentials in the Nts^Ctrl^, Nts^NpHR^, Nos1^Ctrl^, and Nos1^NpHR^ neurons with optical inhibition. Bar graphs illustrating (H) the spike variation, presented as the ratio of firing rates with versus without optical stimulation. Data are mean ± SEM (*n* = 10 neurons from 3 mice in Nts^Ctrl^ and Nts^NpHR^ group, *n* = 11 neurons from 3 mice in Nos1^Ctrl^ and Nos1^NpHR^ group, Nts^Ctrl^ versus Nts^NpHR^ ****P < 0.0001; Nos1^Ctrl^ versus Nos1^NpHR^ ****P < 0.0001, *t* test). (I) Schema of the DNMP T maze with optical inhibition during different phases. The delay phase was set to 60 s. (J to L) Bar graphs showing the performance of Nts^Ctrl^, Nts^NpHR^, Nos1^Ctrl^, and Nos1^NpHR^ mice in the testing sessions of DNMP T maze with optical inhibition during different phases. Optical inhibition was delivered to the cell body by implanting the optical fiber to the MM. Data are mean ± SEM (*n* = 9 per group, Light on: Nts^Ctrl^ versus Nts^NpHR^ **P = 0.0061; Nos1^Ctrl^ versus Nos1^NpHR^ **P = 0.0019, 2-way ANOVA).

The distinct gene expression profiles, cellular properties, and circuitry organization of Nts and Nos1 neurons suggest their specialized functional roles within the MM. To investigate their functional roles, we used rAAV2/9-hSyn-DIO-Kir2.1-EGFP to express the inward-rectifying potassium channel Kir2.1 [31,32], selectively suppressing the activity of Nts and Nos1 neurons (Fig. 6A). Compared to control groups (Nts^Ctrl^ and Nos1^Ctrl^), Kir2.1-expressing neurons (Nts^Kir2.1^ and Nos1^Kir2.1^) exhibited hyperpolarized resting membrane potentials, elevated action potential thresholds, and reduced spontaneous firing (Fig. 6B and C and Fig. S5).

Behavioral assessments encompassing homecage activity, light–dark box, elevated plus maze (EPM), open field, 3-chamber, and Morris water maze tests revealed that functional impairment of neither Nts nor Nos1 neurons affected general motor function, emotional responses, preference behaviors, social memory, or long-term memory (Fig. S6A to F). We then assessed working memory using the DNMP T-maze task. In this task, the mouse was forced to enter either the left or right arm during a sample phase, and a reward was delivered only when it entered the opposite arm during the subsequent choice phase. After the mouse acquired the ability to alternate, the delay between the sample and choice phases was extended to evaluate its memory of the initially visited arm as a measure of spatial working memory. Mice with inhibited Nts or Nos1 neurons performed comparably to controls during training sessions but exhibited significant accuracy deficits in the testing sessions with the delay phase of 30, 60, and 90 s (Fig. 6D and E and Fig. S6G). Notably, performance remained intact when no delay was introduced between sample and choice phases (Fig. S6G), suggesting that Nts and Nos1 neurons are essential for working memory maintenance, specifically by sustaining spatial information encoded during the sample phase throughout the delay phase. Notably, impaired working memory was observed in the DNMP T-maze task, whereas no deficit was detected in the Morris water maze test, although the Morris water maze task also engages working memory components during its initial learning phase. We propose that this discrepancy arises from the differing working memory demands and the distinct nature of memory assessed by each task. In the Morris water maze task, long-term spatial memory is consolidated over multiple days of repeated training, a process that may overshadow or compensate for transient working memory deficits. In contrast, the DNMP T maze specifically taxes working memory on a trial-unique basis, requiring short-term maintenance of spatial information acquired during a single sample phase, rather than relying on long-term memory consolidation.

To further elucidate the temporal-specific role of Nts and Nos1 neurons, we employed phase-selective optogenetic inhibition during testing sessions. Both somatic and axonal inhibition of Nts and Nos1 neurons selectively impaired working memory performance only when applied during the delay phase, supporting their critical role in memory maintenance (Fig. 6F to L and Fig. S7A to D). These findings align with previous reports demonstrating the critical role of the AV, the downstream target of projections from both Nts and Nos1 neurons, in working memory maintenance [33].

**Fig. 7.**
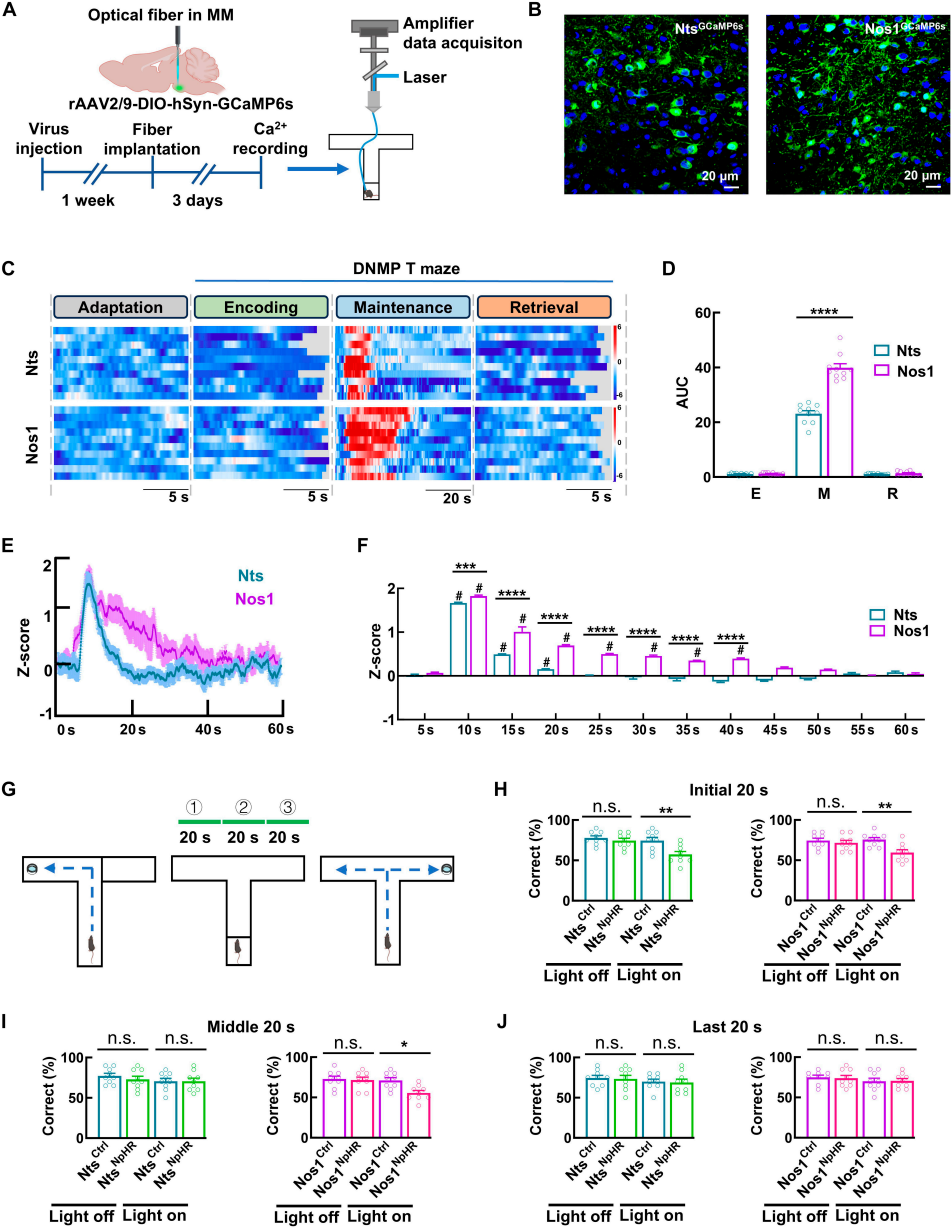
Nts and Nos1 neurons exhibit distinct temporal engagement in working memory maintenance. (A and B) Schematic of Ca^2+^ imaging of Nts and Nos1 neurons during the testing session with correct performance in DNMP T maze (A) and representative images showing the expression of GCaMP6s in Nts and Nos1 neurons (B) by injecting rAAV2/9-hSyn-DIO-GCaMP6s into the MM of Nts^CRE^ and Nos1^CRE^ mice. (C and D) Heat maps (C) and bar graphs (D) showing the neuronal activity of Nts^GCaMP6s^ and Nos1^GCaMP6s^ neurons during the encoding, maintenance (60 s of delay phase), and retrieval of working memory of DNMP T maze. Data are mean ± SEM (*n* = 10 mice per group, Nts versus Nos1 ****P < 0.0001, 2-way ANOVA). (E and F) Z-score of Ca^2+^ dynamics (E) and bar graphs (F; time binned at 5 s) showing the temporal neuronal activity of Nts^GCaMP6s^ and Nos1^GCaMP6s^ neurons during the working memory maintenance (60 s of delay phase) of DNMP T maze. Data are mean ± SEM (*n* = 10 mice per group; # indicates a within-group difference compared to the baseline, ^#^*P* < 0.05, *t* test; * indicates a between-group difference, ***P = 0.0003, ****P < 0.0001, 2-way ANOVA). (G) Schema of the DNMP T maze with optical inhibition during the 3 (initial, middle, and last) 20-s periods within the 60-s delay phases of DNMP T maze. (H to J) Bar graphs showing the performance of Nts^Ctrl^, Nts^NpHR^, Nos1^Ctrl^, and Nos1^NpHR^ mice in the testing sessions of DNMP T maze with optical inhibition the during the three 20-s periods within the 60-s delay phases. Optical inhibition was delivered to the cell body by implanting the optical fiber to the MM. Data are mean ± SEM (*n* = 9 mice per group, Initial 20 s Light on Nts^Ctrl^: Nts^NpHR^ **P = 0.0056, Light on Nos1^Ctrl^ versus Nos1^NpHR^ **P = 0.0050, Middle 20 s Light on Nos1^Ctrl^ versus Nos1^NpHR^ *P = 0.0140, 2-way ANOVA).

We further investigated functional differences between Nts and Nos1 neurons by monitoring their neuronal activity through GCaMP6s-mediated Ca^2+^ imaging (Fig. 7A and B). During the testing session with correct performance, both neuron types became selectively active at delay phase onset, with activity gradually declining thereafter. Notably, Nos1 neurons exhibited sustained activation for approximately 40 s—twice as long as Nts neurons (~20 s)—regardless of total delay duration (Fig. 7C to F). This temporal difference was further confirmed through optogenetic manipulation across segmented delay phases (three 20-s periods during the 60-s delay phase). Inhibition of either Nts neurons or their projections significantly impaired performance only when applied during the initial 20 s, whereas somatic or axonal inhibition of Nos1 neurons caused performance deficits during both initial and middle 20-s periods (Fig. 7G to J and Fig. S7E to G).

### *Grik4* is involved in working memory maintenance and contributes to working memory deficits in early AD

**Fig. 8.**
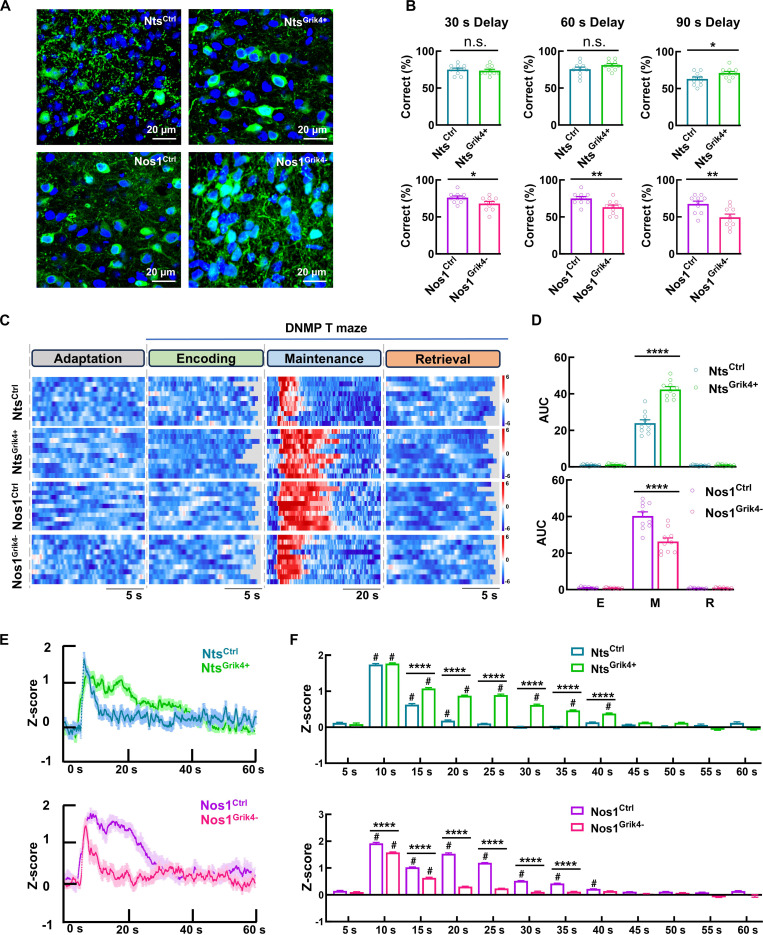
*Grik4* mediates the neuronal activity difference of Nts and Nos1 neurons in working memory maintenance. (A) Representative images showing the expression of GCaMP6s with or without *Grik4* overexpression in Nts neurons (Nts^Ctrl^ and Nts^Grik4+^) and knockdown in Nos1 neurons (Nos1^Ctrl^ and Nos1^Grik4−^) by respectively injecting rAAV2/9-hSyn-DIO-*Grik4*-GCaMP6s (rAAV2/9-hSyn-DIO-GCaMP6s as control) and rAAV2/9-hSyn-DIO-GCaMP6s-shRNA-*Grik4* (rAAV2/9-hSyn-DIO-GCaMP6s-shRNA-scramble as control) into the MM of Nts^CRE^ and Nos1^CRE^ mice. (B) Bar graphs showing the performance of Nts^Ctrl^, Nts^Grik4+^, Nos1^Ctrl^, and Nos1^Grik4−^ mice in the testing sessions of DNMP T maze with 30-, 60-, and 90-s delay phase. Data are mean ± SEM (*n* = 10 mice per group, 30 s Delay Nos1^Ctrl^ versus Nos1^Grik4−^ *P = 0.0419, 60 s Delay Nos1^Ctrl^ versus Nos1^Grik4−^ **P = 0.0072, 90 s Delay Nts^Ctrl^ versus Nts^Grik4+^ *P = 0.0344, Nos1^Ctrl^ versus Nos1^Grik4−^ **P = 0.0058, *t* test). (C and D) Heat maps (C) and bar graphs (D) showing the neuronal activity of Nts^Ctrl^, Nts^Grik4+^, Nos1^Ctrl^, and Nos1^Grik4−^ neurons during the encoding, maintenance (60 s of delay phase), and retrieval of working memory of DNMP T maze. Data are mean ± SEM (*n* = 10 per group, Nts^Ctrl^ versus Nts^Grik4+^ ****P < 0.0001; Nos1^Ctrl^ versus Nos1^Grik4−^ ****P < 0.0001, *t* test). (E and F) Z-score of Ca^2+^ dynamics (E) and bar graphs (F, time binned at 5 s) showing the temporal neuronal activity of Nts^Ctrl^, Nts^Grik4+^, Nos1^Ctrl^, and Nos1^Grik4−^ neurons during the working memory maintenance (60 s of delay phase) of DNMP T maze. Data are mean ± SEM (*n* = 10 mice per group; # indicates a within-group difference compared to the baseline, ^#^*P* < 0.05, *t* test; * indicates a between-group difference, ****P < 0.0001, 2-way ANOVA).

Given that both Nts and Nos1 neurons receive excitatory inputs from VS pyramidal neurons with similar presynaptic properties (Figs. 2E to H and 4), we investigated potential molecular mechanisms underlying their differential temporal dynamics during working memory maintenance [34]. The observed high *Grik4* expression in Nos1 neurons, associated with enhanced AMPAR-mediated responses and synaptic plasticity (Fig. 3), prompted us to examine its functional role. Manipulation of *Grik4* in both Nts and Nos1 neurons did not affect motor function, emotional responses, preference behaviors, social memory, or long-term memory (Fig. S8A to F). However, in the DNMP T maze, *Grik4* overexpression in Nts neurons prolonged their activation duration in the delay phase and improved working memory performance, an effect that reached statistical significance only in the 90-s delay paradigm (potentially due to a ceiling effect in the 30- and 60-s paradigms) (Fig. 8B). Meanwhile, *Grik4* knockdown in Nos1 neurons shortened their activity and impaired task accuracy, establishing *Grik4* as a key molecular determinant of the distinct temporal contributions of Nts and Nos1 neurons to working memory maintenance (Fig. 8 and Figs. S8G and S9).

**Fig. 9.**
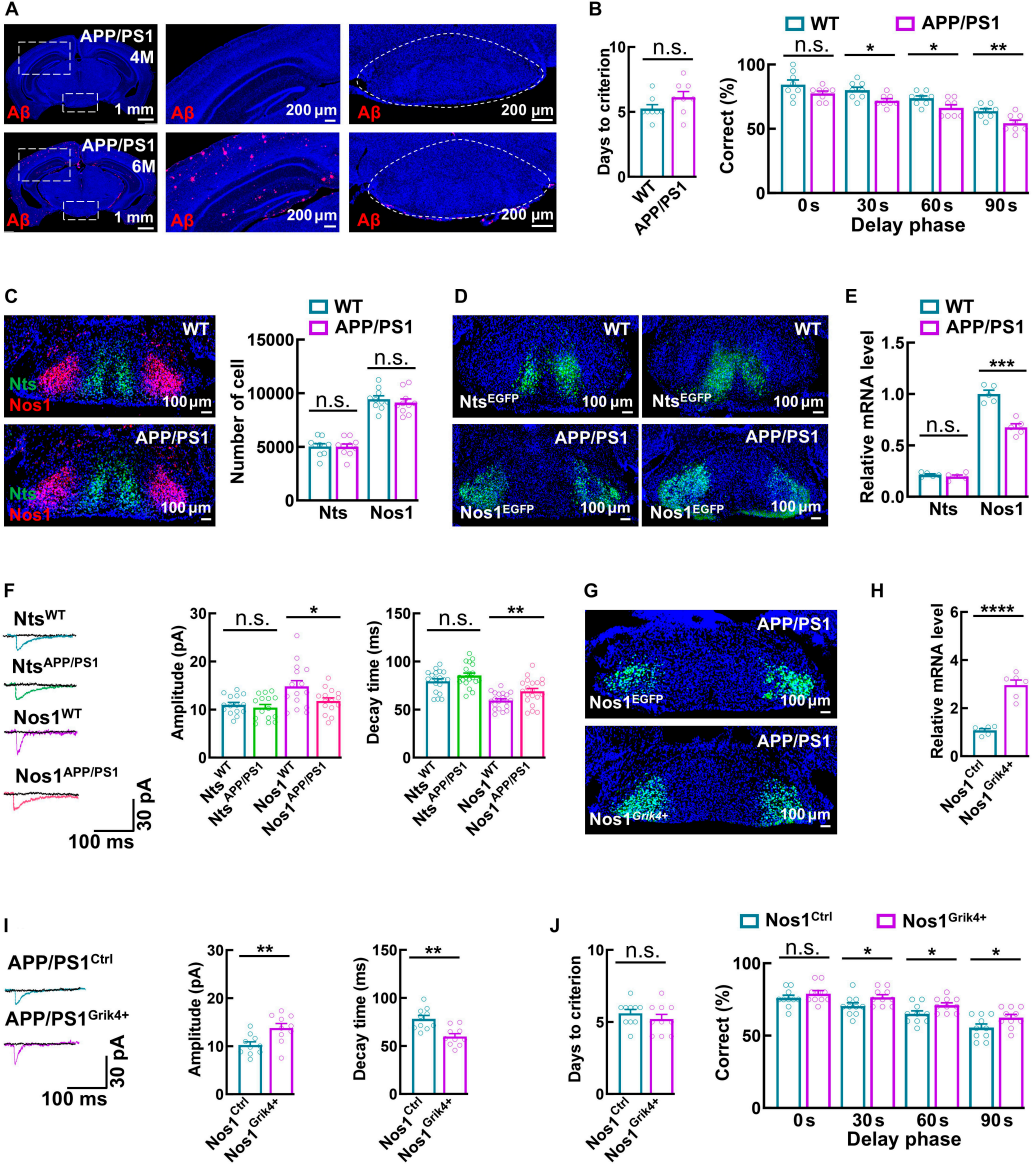
The role of *Grik4* in the early stage of AD. (A) Representative images showing the deposition of Aβ amyloid protein in different brain regions of APP/PS1 mice at 4 and 6 months of age. (B) Bar graphs showing days to criterion and the performance in the DNMP T maze task with different delay phases of wild-type (WT) and APP/PS1 mice at 4 months of age. Data are mean ± SEM (*n* = 8 mice per group, *P = 0.0122 in 30 s, *P = 0.0281 in 60 s, **P = 0.0077 in 90 s, *t* test). (C) Representative images and a bar graph showing the distribution and number of Nts and Nos1 neurons in WT and APP/PS1 mice. Data are mean ± SEM (*n* = 10 mice per group, *t* test). (D) Representative images illustrating the virus-labeled Nts neurons and Nos1 neurons in the MM of WT and APP/PS1 mice. (E) Bar graph showing the mRNA level of *Grik4* (qPCR) in Nts and Nos1 neurons of WT and APP/PS1 mice. Data are mean ± SEM (*n* = 5 mice per group; ***P = 0.0002, *t* test). (F) Representative traces and bar graphs illustrating KAR-mediated EPSCs in Nts and Nos1 neurons of WT and APP/PS1 mice, along with their amplitude and decay time. The currents were blocked by the KAR/AMPA receptor antagonist CNQX (black traces). Data are mean ± SEM (*n* = 15 neurons from 3 mice per group, Amplitude: *P = 0.0347, Decay time: **P = 0.0096, *t* test). (G and H) Representative images and a bar graph showing the virus-labeled Nos1 neurons and relative Grik4 mRNA levels using qPCR after overexpression in Nos1 neurons in APP/PS1 mice at 4 months age. Data are mean ± SEM (*n* = 6 per group; ****P < 0.0001, *t* test). (I) Representative traces and bar graphs illustrating KAR-mediated EPSCs in mice, along with their amplitude and decay time. The currents were blocked by the KAR/AMPA receptor antagonist CNQX (black traces). Data are mean ± SEM (*n* = 10 neurons from 3 mice per group, Amplitude: **P = 0.0064, Decay time: **P = 0.0010, *t* test). (J) Bar graphs showing days to criterion and the performance of mice in the DNMP T maze with different delay phases. Data are mean ± SEM (*n* = 10 mice per group; *P = 0.0487 in 30 s, *P = 0.0372 in 60 s, *P = 0.0485 in 90 s, *t* test).

The MB has been implicated in the early progression of AD [11,12]. To explore the potential medical relevance of our physiological findings, we examined whether the properties of Nts and Nos1 neurons are altered in an AD context. Using APP/PS1 mice, a model of amyloid pathology, at 4 months of age—an age at which they exhibit working memory deficits without overt plaque deposition [35]—we found no changes in the number of Nts or Nos1 neurons in the MM relative to age-matched wild-type controls (Fig. 9A to C). However, Nos1 neurons displayed a selective reduction in *Grik4* RNA levels, which was associated with corresponding changes of KAR-mediated EPSCs. This effect was not observed in Nts neurons (Fig. 9D to F). Importantly, targeted overexpression of *Grik4* in Nos1 neurons rescued both the electrophysiological and behavioral impairments (Fig. 9G to J). These findings suggest that down-regulation of *Grik4* in Nos1 neurons in the MM contributes to working memory deficits in the early stage of AD.

## Discussion

While contemporary research on the MB and associated pathologies has made substantial advances [1,11,28,36,37], knowledge gaps remain at cellular and circuit levels. Two particularly understudied areas demand urgent investigation: (a) systematic classification of neuronal types within this complex region, and (b) characterization of neuron type-specific circuit organization and its functional contributions to memory processes. In this study, we employed multimodal approaches to systematically investigate Nts and Nos1 neurons across multiple scales. Integration of these findings with our previous work on PV and Drd2 neurons [17] and recent studies about the MB [38,39] reveals a clear hierarchical organization of the MB—from molecular profiles to cellular identities, circuit connectivity, and functional properties.

The Papez circuit, a well-characterized closed-neural pathway studied for nearly a century, integrates the hippocampus, thalamus, and cortex to mediate memory and emotion [40,41]. Within this circuit, the MBs act as a relay hub between 2 key memory-related regions: the hippocampus and the anterior thalamic nucleus [36]. However, the neuronal type-specific circuitry and functional mechanisms remain unclear. Here, we demonstrate that both Nts and Nos1 neurons primarily receive excitatory inputs from the VS and send parallel excitatory projections to the dmAV and vlAV (dorsomedial and ventrolateral subdivisions of the AV), 2 newly molecularly defined subregions [29]. Strikingly, unilateral VS innervates Nts and Nos1 neurons bilaterally, whereas unilateral Nts and Nos1 neurons project only ipsilaterally to the AV. Furthermore, we found that Nts and Nos1 neurons—functionally aligned with downstream AV [33]—are essential for the maintenance of working memory. Both types of neurons are selectively activated at the onset of the delay phase that gradually declines over time. Notably, due to high *Grik4* (glutamate ionotropic receptor kainate type subunit 4) expression, the activation duration of Nos1 neurons was approximately twice as long as that of Nts neurons, reaching up to ~40 s, while the activation duration of Nts neurons is about 20 s. Extending these findings, we discovered that the decrease of Grik4 in Nos1 neurons governs the working memory deficit in the early stage of AD (APP/PS1 mice at 4 months age).

KA1, a high-affinity subunit of the KAR, is encoded by *Grik4* and highly expressed in Nos1 neurons. While KA1 predominantly interacts with other KAR subunits (encoded by *Grik1*/*2*/*3*/*5*) to form functional glutamate-gated KARs, none of these subunits showed differential expression between Nts and Nos1 neurons (Fig. 3A). This suggests that the observed differences in KAR properties and related functions between Nts and Nos1 neurons are specifically attributable to KA1-containing KARs.

During the maintenance of memory, including working memory, the number of participating neurons and their activation levels exhibit a progressive decline. This progressive decline in both the number and activation level of engaged neurons mechanistically explains the observed behavioral correlation: Longer delay phases result in decreased accuracy [42–44]. Within this framework, the distinct activation profiles of Nts and Nos1 neurons—the former limited to the first 20 s of the maintenance phase, the latter persisting for up to approximately 40 s—offer a suitable neuronal correlate of memory degradation. According to the neuronal drift hypothesis of working memory, memory precision decays over time due to drift in neuronal activity patterns [45]. The cessation of Nts neuronal activity at around 20 s may reflect a loss of information, while the sustained activity of Nos1 neurons may counteract such drift by preserving information across the delay. Together, Nts-mediated flexibility for updating and Nos1-mediated stability for retention enable the memory system to balance rapid updating with resistance to temporal decay, supporting performance in dynamic contexts.

In addition to the VS, the VTg also projects to Nts and Nos1 neurons with a unilateral-to-bilateral pattern, although these projections are inhibitory [30]. The VTg is reciprocally interconnected with the MM, and the VTg neurons are thought to discharge rhythmically with a high degree of coherence to the hippocampal theta rhythm [46]. Both the VS and VTg are critical for modulating theta rhythm in downstream brain regions, a rhythm essential for working memory. Thus, we propose that the VS and VTg may sustain working memory through coordinated regulation of the theta rhythm in the MM.

In addition to AD and other cognitive disorders, clinical studies have shown that in conditions of global cerebral hypoxia, such as neonatal hypoxic–ischemic encephalopathy, the MBs exhibit early and selective vulnerability, with damage occurring earlier than in other brain regions. Our current and previous electrophysiological recordings in vitro have consistently demonstrated that MB neurons display exceptionally high firing activity, exceeding 200 Hz, which is markedly greater than that of classical PV-positive fast-spiking interneurons (typically around 100 Hz) [47]. We hypothesize that such hyperexcitability substantially elevates neuronal energy and oxygen demands, rendering MB neurons more susceptible to injury during hypoxic–ischemic events. This may underlie their selective involvement in cerebral hypoxic–ischemic disorders. As a critical relay hub within the Papez circuit—a pathway integral to both emotion and cognition—damage to the MB is likely to disrupt Papez circuit function, contributing to the memory and emotional deficits commonly observed following global hypoxic insults. Of course, the specific vulnerability of the MB to global cerebral hypoxia cannot exclude the possibility of interregional differences in cerebral blood supply, meaning that the MB may receive relatively lower blood flow compared to other brain regions. Our findings not only provide new insights into the connectivity and functional organization of the Papez circuit but also reveal novel molecular, cellular, and circuit-level mechanisms governing working memory processes.

In conclusion, our cross-scale investigation of Nts and Nos1 neurons in the MB—spanning molecular to functional levels—has enriched the anatomical and functional characterization of both the MB and Papez circuit, while advancing our understanding of working memory mechanisms (Fig. S10). Importantly, these findings support a comprehensive basis for exploring therapeutic interventions targeting MB-associated memory disorders, particularly AD.

In this study, we demonstrated that pyramidal neurons from the unilateral VS project into the MB via an ipsilateral axonal pathway and bilaterally innervate Nts and Nos1 neuronal subgroups with the axon distributed along the horizontal axis of MB in mice (Fig. S3A). However, this observed connectivity pattern may not be conserved in primates (including humans), given that their MBs exist as anatomically separated, paired, and symmetrical subnuclei, in contrast to the singular, unified structure observed in rodents. To address these potential interspecies differences of the VS-MB projection, anterograde/retrograde neural circuit tracing methodologies should be further employed to investigate the VS-MB connectivity patterns (unilateral-to-ipsilateral, unilateral-to-contralateral, or unilateral-to-bilateral) in primate species, including human.

## Methods

### Animals

The C57BL/6J mice and Nts^CRE^ transgenic mice were obtained from Shanghai Model Organisms (Shanghai, China), while the Nos1^CRE^ transgenic mice were acquired from Cyagen Biosciences Inc. (Guangzhou, China). APP/PS1 (stock no. 034832) were purchased from the Jackson Laboratory. Male mice were exclusively used to eliminate sex-based variability. All mice were housed under standardized conditions at Huazhong University of Science and Technology (Wuhan, China), following institutional guidelines and with approval from the facility’s Animal Care and Use Committee. The animals were housed in groups of 3 to 5 per cage. The housing environment was strictly controlled, with a 12-h light/dark cycle (lights on at 08:00), a constant temperature of 22 °C, and a relative humidity of 50 ± 5%. Behavioral testing was performed during the light cycle after a 7-d habituation period. Subjects underwent stratified randomization across experimental groups using previously established methodology [48,49]. All experiments were performed following the guidelines of the National Natural Science Foundation of China and approved by the Animal Care and Use Committee of the animal core facility at Huazhong University of Science and Technology, Wuhan, China.

### Virus and injection

For labeling Nts and Nos1 neurons, rAAV2/9-hSyn-DIO-EGFP was injected into the MM of Nts^CRE^ and Nos1^CRE^ mice. rAAV2/9-hSyn-DIO-mCherry-TVA-G, rAAV2/9-hSyn-mCherry-TVA-G, and EnvA-RV(CVS-N2C)-ΔG-EGFP were used for monosynaptic retrograde tracing of Nts and Nos1 neurons in the MM and the neurons in the AV. rAAV2/9-hSyn-tdTomato and rAAV2/9-hSyn-EGFP were used to label the neurons from unilateral DS and VS. rAAV2/2-retro-hSyn-Flpo and rAAV2/9-hSyn-Con/Fon-EGFP were used to unilaterally target Nts and Nos1 neurons in the MM. rAAV2/2-retro-hSyn-DIO-Flpo, rAAV2/9-fDIO-mRuby3-TVA-G, and EnvA-RV(CVS-N2C)-ΔG-EGFP were used for monosynaptic retrograde tracing of unilateral Nts and Nos1 neurons in the MM. For calcium imaging of Nts and Nos1 neurons, rAAV2/9-hSyn-DIO-GCaMP6s was applied. To overexpress *Grik4* in Nts neurons with or without calcium imaging, rAAV2/9-hSyn-DIO-*Grik4*-EGFP and rAAV2/9-hSyn-DIO-*Grik4*-GCaMP6s were injected into the MM of Nts^CRE^ mice, while rAAV2/9-hSyn-DIO-EGFP and rAAV2/9-hSyn-DIO-GCaMP6s were used as control. To knock down *Grik4* in Nos1 neurons, we injected rAAV2/9-hSyn-DIO-EGFP-shRNA-*Grik4* and rAAV2/9-hSyn-DIO-GCaMP6s-shRNA-*Grik4* (rAAV2/9-hSyn-DIO-EGFP-shRNA-scramble and rAAV2/9-hSyn-DIO-GCaMP6s-shRNA-scramble as control) into the MM of Nos1^CRE^ mice. To inhibit the neuronal activity, rAAV2/9-hSyn-DIO-Kir2.1-EGFP was applied to express Kir2.1 in Nts and Nos1 neurons of the MM. For optogenetic manipulation, we applied rAAV2/2-retro-hsyn-flpo and rAAV2/9-hSyn-Con/Fon-ChR2(H134R)-EGFP to express ChR2 in the unilateral Nts and Nos1 neurons of the MM, rAAV2/9-hsyn-ChR2(H134R)-tdTomato in the neurons of unilateral VS, and rAAV2/9-hSyn-DIO-eNpHR3.0-EGFP in Nts and Nos1 neurons of the MM. All viral vectors were packaged by Taitool Bioscience, except for RNA interference-related recombinant adeno-associated viruses (rAAVs) (shRNA-*Grik4*: AATTCCTTATCTCCAAGGACA), which were provided by BrainVTA.

For viral injection, after weighing, mice were anesthetized via intraperitoneal injection of 2% sodium pentobarbital (50 mg/kg). Once fully anesthetized, the mouse’s head was fixed in a stereotaxic adapter. The scalp was disinfected with alcohol and surgically opened to expose the skull and sutures. Target coordinates were marked according to the bregma [MM: mediolateral (ML) 0 mm, anteroposterior (AP) −2.8 mm, dorsoventral (DV) 5.7 mm; AV: ML ±1.0 mm, AP −0.7 mm, DV 3.2 mm; VS: ML ±2.75 mm, AP −3.6 mm, DV 5.4 mm; DS: ML ±1.7 mm, AP −3.1 mm, DV 1.6 mm]. The needle was maintained in situ for 30 min post-injection before slow withdrawal.

rAAVs (diluted to 5 × 10^12^ particles/ml) were microinjected at a volume of 300 nl per site. Behavioral tests, optogenetic manipulations, and calcium imaging were performed 14 d post-injection to allow for sufficient transgene expression. For monosynaptic retrograde tracing, RV (400 nl of 1.8 × 10^8^ particles/ml) was microinjected 14 d after initial expression of TVA and G protein. Imaging experiments were conducted following an additional 14-d incubation period post-RV injection.

### Immunofluorescence and FISH

Mice underwent anesthesia via intraperitoneal pentobarbital sodium overdose followed by transcardial perfusion with 150 ml of saline (0.9% w/v NaCl) and 4% paraformaldehyde (PFA). Extracted brains were postfixed in 4% PFA for 24 h prior to sectioning into 30-μm coronal slices (Leica Microsystems, Wetzlar, Germany). In brief, staining was performed on 30-μm coronal sections. The sections were subsequently incubated in tris–HCl buffer (50 × 10^−3^ M) supplemented with 3% donkey serum and 0.3% Triton X-100. Brain sections were then incubated with antibodies from 2 different species at 4 °C 24 h for immunofluorescence labeling. The following antibodies were used in this study: mouse anti-CaMKIIα (1:400, Proteintech, 66843-1-Ig), guinea pig anti-GAD1 (1:300, SYSY, 198 308), rabbit anti-PCP4 (1: 300, Proteintech, 14705-1-AP), and mouse anti-Calb1 (1: 300, Swant, CB300). Following phosphate-buffered saline (PBS) rinses, sections were incubated with fluorophore-conjugated secondary antibodies (Thermo Fisher) at room temperature for 90 min. After final washes, sections were dehydrated and mounted with fluorescence mounting medium (Invitrogen, P36961).

For FISH, 20-μm-thick coronal brain sections were cut. Sections containing the MB were identified and mounted on Superfrost Plus slides (Epredia, J1800AMNZ) and stored at −80 °C in sealed slide boxes until hybridization procedures. FISH probes targeting Nts (420441-C1), Nos1 (437651-C2), PV (421931-C3), Drd2 (406501-C4), Slc17a6 (319171-C3), and Slc32a1 (319191-C3) were procured from Advanced Cell Diagnostics (ACD; Hayward, CA), along with ancillary hybridization reagents. All experimental procedures including antigen retrieval, protease pretreatment, target hybridization, signal amplification, and chromogenic detection were strictly implemented according to the ACD RNAscope Protocol (323100). The labeled specimens were imaged using an FV3000 FLUOVIEW confocal laser scanning microscope, followed by 3-dimensional (3D) construction analysis with Image-Pro Plus software. Z-series imaging was conducted at 0.5-μm axial intervals across target regions using a 20× U Plan XApochromat objective [numerical aperture (NA) 0.8, working distance (WD) 0.6 mm], with 8 to 12 optically sectioned images compiled into z-stacks for visualization purposes. We quantified the absolute numbers of single, double, or triple labeled cells by sampling every section (image stacks) from the experimental animals, as described before [17]. Reconstruction of the complete sample architecture was achieved by maximum-intensity projection and stitching of all z-stack images. For manual quantification, analysis was performed on 20× magnification images by employing the ImageJ Cell Counter plugin to assess colocalization.

### Population cell mRNA-seq

Targeted isolation of Nts and Nos1 neurons from the MM was achieved through whole-cell patch-clamp in acute brain slices (*n* = 4 mice per group across 3 independent litters). Tissue dissociation was employed in buffer containing 10 mM tris–Cl (pH 7.6), 50 mM NaF, 1 mM Na_3_VO_4_, 1 mM edetic acid, 1 mM benzamidine, 1 mM phenylmethylsulfonyl fluoride (PMSF), 1 mg/10 ml papain, and a mixture of aprotinin, leupeptin, and pepstatin A (10 mg/ml each) for 30-min enzymatic digestion at room temperature. Suspended Nts^EGFP^ or Nos1^EGFP^ neurons were flow-sorted using the S3e Cell Sorter (Bio-Rad), followed by homogenization and dilution with ice-cold RNAlater solution (Sigma-Aldrich, #R0901). Single-cell lysis was conducted in 1 μl of buffer containing RNasin inhibitor, followed by mRNA–cDNA conversion via oligo-dT primed reverse transcription. PCR-amplified cDNA underwent Tn5 transposase-mediated fragmentation, bead-purified, and subjected to Illumina library construction. PCR products were purified with Agencourt AMPure XP beads (Beckman Coulter A63880) and eluted in EB buffer (Qiagen 19086). cDNA quality control was conducted using the Agilent 2100 BioAnalyzer with High Sensitivity DNA Kit (Agilent 5067-4626). Sequencing libraries were prepared with Nextera XT DNA Library Prep Kit (Illumina FC-131-1024), followed by sequencing on Illumina NovaSeq (>1 million reads per sample). TopHat2 (v2.1.0) aligned reads to mouse mm10 genome with default parameters for mapping rate calculation. Gene expression quantification was performed via RSEM (v1.2.8) using Bowtie2 (v2.2.9)-aligned reads against UCSC gene annotations. Exonic reads were enumerated by featureCounts (Subread v1.5.1) for reads per kilobase per million mapped reads (RPKM) calculation. Final dataset included 224 neurons (110 Nts^+^/114 Nos1^+^) with median 10,092 genes per cell. Differential gene expression analysis was conducted through the edgeR (v3.12.1) with dual selection criteria: absolute fold change ≥ 2 and significance threshold of *P* < 0.05.

### Slice preparation and electrophysiology

Electrophysiological recordings were obtained from neurons (4 to 6 mice per group, 2 to 3 slices per mouse, 3 to 5 cells per slice). The slices were prepared and transferred to a holding chamber that contained artificial cerebrospinal fluid (ACSF; 124 mM NaCl, 3 mM KCl, 26 mM NaHCO_3_, 1.2 mM MgCl_2_, 1.25 mM NaH_2_PO_4_2H_2_O, 10 mM C_6_H_12_O_6_, and 2 mM CaCl_2_ at pH 7.4, 305 mOsm) at 32 °C for 30 min. The slices were transferred to a recording chamber, which was continuously perfused with oxygenated ACSF (2 ml/min) at room temperature for 60 min. Nts and Nos1 neurons were visualized using an Axioskop 2FS upright microscope equipped with infrared–phase-contrast (IR-DIC) optics and a Hamamatsu C2400-07E camera, as previously described [50], and the morphology of Nts and Nos1 neurons was confirmed by filling the neurons with biocytin and then visualized with streptavidin [51]. Z-series imaging of labeled neurons was performed using the FV3000 FLUOVIEW confocal laser scanning microscope, followed by 3D reconstruction and analysis of the imaging data with Imaris software.

Intracellular recordings were obtained from genetically identified Nts^EGFP^ and Nos1^EGFP^ neurons in the MM. For voltage-clamp recordings, the intracellular solution contained the following: 140 mM potassium gluconate, 0.05 mM EGTA, 10 mM Hepes, 2 mM Mg-ATP (adenosine triphosphate), 0.2 mM Na-GTP (guanosine triphosphate), 140 mM potassium gluconate, 0.05 mM EGTA, 10 mM Hepes, 2 mM Mg-ATP, and 0.2 mM Na-GTP. Resting membrane potentials were acquired within 2 min of membrane rupture, with steady-state potentials clamped at −65 mV or −90 mV through current injection to investigate tonic and burst firing modes, respectively. Action potential thresholds were operationally defined as the membrane potential corresponding to a depolarization rate exceeding 10 V/s. To characterize repetitive spiking patterns in both tonic and burst firing modes, a series of positive current steps were applied (amplitude range: 50 to 300 pA, in 50-pA increments; duration: 500 ms). The firing frequency was measured during the initial 100 ms of spiking, while the number of action potentials was counted within the first 500 ms following a hyperpolarizing stimulus.

To record spontaneous activity from the Nts and Nos1 neurons, the internal solution consisted of 140 mM potassium gluconate, 0.05 mM EGTA, 10 mM Hepes, 2 mM Mg-ATP, and 0.2 mM Na-GTP. The perfusion solution was supplemented with picrotoxin (PTX) (100 μM), AP5 (50 μM), and LY303070 (25 μM) to isolate KAR-mediated currents. To record mEPSCs, TTX (1 μM) was added to the solution. The AMPAR/KAR antagonist 6-cyano-7-nitroquinoxaline-2,3-dione (CNQX) was added to the recording solution as required. To calculate the AMPAR /NMDAR current ratio, AMPA currents were recorded at −65 mV in the presence of PTX (100 μM) and were fully blocked by CNQX (10 μM). Subsequently, NMDA currents were measured at +40 mV, and their identity was confirmed by reversible blockade with AP5 (100 μM). The neurons were first clamped at −65 mV for 5 min, and the holding potential was then switched to +40 mV for an additional 5 min. Each evoked response was repeated 30 times with an interstimulus interval of 10 s, and they were averaged.

When we test the optogenetically evoked synaptic responses, we use the intracellular solution: 120 mM CsCH_3_SO_3_, 20 mM CsCl, 4 mM Nacl, 10 mM Hepes, 4 mM Mg-ATP, 3 mM QX314, 0.05 mM EGTA, and 0.2 mM Na-GTP. Synaptic currents in neurons were evoked by blue laser light stimulation (2 ms of 473-nm light at an intensity of ~5 mW/cm^2^, DPSS laser, Inper Co. Ltd., Hangzhou, China). Laser light was delivered via a 40× water immersion objective (collimation and reflection system), producing a 300-μm focal spot with 29.1-mW maximum power at the focal plane. EPSCs were blocked by TTX (1 μM), rescued by 4-aminopyridine (4-AP; 100 μM), and reversed by AP5 (50 μM). We established 18 repeated light stimulation sweeps in each single cell recording (60-s duration per sweep, with fixed parameter photostimulation delivered at consistent time points in each sweep) and selected neurons that exhibited reliable light-evoked postsynaptic responses in every sweep. The mean amplitude of these responses was recorded. Under identical recording conditions in the absence of light stimulation, we also measured the baseline spontaneous postsynaptic currents and defined the average of this baseline as the “response current threshold”. Recording sites were determined by randomly selecting microscopic fields, and neurons within those fields were subsequently recorded. For statistical analysis, only neurons that showed consistent light-evoked postsynaptic currents in all 18 sweeps, with amplitudes consistently exceeding the response current threshold, were included.

We measured field excitatory post-synaptic potentials (fEPSPs) using extracellular recordings. Data were monitored online, and EPSP slopes were measured by taking the slope of the rising phase between 5% and 95% and analyzed off-line using Win LTP software, as described before [48]. Following 10-min baseline recording of 0.1 Hz-evoked fEPSPs, LTP saturation was induced via high-frequency stimulation (HFS; 100 Hz, 1 s) with subsequent 40-min response monitoring. Stimulation intensity was calibrated to 35% of maximum synaptic response during baseline. Electrophysiological signals underwent 2-kHz low-pass filtering and digital conversion (10 to 20 kHz sampling rate) using a Digidata 1440A digitizer (Molecular Devices). Series resistance (Rs) was actively stabilized, with data exclusion criteria set at >20% Rs variation during recordings. KAR and AMPA receptor currents were measured before and after LTP induction in MB slices obtained from the same cohort of mice. To minimize potential confounders such as drug washout residual effects and interindividual variability, we used separate slices from the same mice for pre- or post-LTP recordings. To calculate the ratio of KAR/AMPAR-mediated currents before LTP/after LTP, we compared the values recorded from different brain sections of the same mouse.

Under bright-field microscopy, the target brain region was centered. Using the visualization device, we observed neuronal morphology thoroughly and selected neurons with clear particles and appropriate cell size. The electrode was positioned directly above the target cell. Once the electrode tip contacts the cell, indicated by a resistance increase of 0.1 to 0.2 MΩ, gentle negative pressure was applied via the connected syringe to attract the neuron and initiate sealing. The electrode resistance was monitored on the system screen. When resistance stabilizes at the GΩ level, 2 min was allowed for cell stabilization. Then, the syringe was opened and continuous negative pressure was slowly applied to rupture the membrane. After successful rupture, we confirmed on the monitoring software that the resistance was below 30 MΩ and leak current did not exceed 100 pA. The preparation was allowed to stabilize for 2 to 3 min before the experimental recording began. The electrophysiological signals were low-pass filtered at 2 kHz and digitized at a sampling rate of 10 to 20 kHz with a Digidata 1440A digitizer (Molecular Devices). Rs was actively stabilized, with data exclusion criteria set at >20% Rs variation during recordings.

### Home cage behaviors

Mice were individually housed in PhenoTyper home chambers [40 cm (*L*) × 40 cm (*W*) × 40 cm (*H*); Noldus] to evaluate natural exploratory motivation and repetitive behaviors with ad libitum resources under 12/12-h light–dark cycles. Following 24-h acclimation, spontaneous behaviors were monitored for 48 h via infrared beam arrays, with automated EthoVisionXT (Noldus) video analysis quantifying exploratory and stereotypic patterns as described before [50].

### Open field test

Behavioral testing was measured in an standardized chamber (50 cm × 50 cm × 40 cm). Mice underwent 30-min environmental habituation in the testing room prior to the test. Spontaneous motor activity of mice was recorded for 5 min. Individual mice were placed in the center of the chamber at the beginning of the test. The central area of the chamber was defined as 50% of the open field arena. The time spent in the central area, the time spent in the corner area, and the total distance moved of mice were recorded by EthoVision XT15 (Noldus, Wageningen, Netherlands).

### EPM test

Behavioral testing was conducted using a standardized murine EPM apparatus positioned 50 cm above floor level. The 4-arm configuration comprised 2 opposing closed arms (15 cm high protective walls) and 2 open arms (0.5 cm raised edges), with all arms maintaining uniform dimensions (63 cm length × 6 cm width). Mice were placed at the central of the EPM and allowed free exploration for 5 min. Behavioral recordings were terminated immediately after the session, and the animals were returned to their home cages. Open arm entries (full-body transitions), open arm dwell time, and closed arm dwell time were quantified using an automated video tracking system.

### Light/dark box test

The light–dark box apparatus consisted of 2 adjacent chambers (20 cm × 20 cm × 25 cm each) constructed with opaque polyvinyl chloride. A rectangular gate (6 cm width × 7 cm height) permitted free passage between the light chamber and dark chamber. The dark chamber was clad with black anodized aluminum panels to achieve full-spectrum attenuation, while the light chamber maintained natural photopic illumination. Mice were placed in the illuminated chamber and allowed free exploration for 5 min. Path length, velocity, and zone transition frequency were quantified by Noldus EthoVision XT15 (Noldus, Wageningen, Netherlands).

### Morris water maze test

Morris water maze was conducted in a 1.5-m-diameter basin filled with nontoxic white ink water (25.0 ± 0.5 °C). Mice underwent 30-min environmental habituation in the testing room prior to the test for 1 to 2 d. The 6-d training protocol consisted of 5 daily trials with randomized entry points. During initial training (day 1), mice received 30-s platform habituation followed by a 60-s free navigation period for searching the platform. Mice that failed to find the platform within this time were guided to it and allowed to remain for 30 s. Following training, mice underwent a single probe trial. Swimming path characteristics (trajectory patterns, quadrant-specific latency, and annulus crossings) were quantified using an automated behavioral tracking system (Techman WMT-100S, China). During the training sessions, each mouse completed 4 trials, with release locations randomized from 4 different starting points in the pool. Following training, probe trials were conducted during the test session. All behavioral assessments were performed by experimenters blinded to genotype and treatment conditions.

### DNMP T maze

Spatial working memory assessment employed the DNMP T maze. Subject mice underwent controlled food restriction until attaining 85% of the free-feeding body weight. During this acclimation phase, mice were progressively familiarized with 14 mg of sucrose reward pellets (14 mg, Bio-Serv, F05684), which would subsequently be used as a reward in the T maze.

Mice were habituated to the T maze for 10 min. Maze arms were sanitized with 70% ethanol solution, and sucrose reward pellets were immediately replenished following consumption. The training sessions comprised 10 daily trials conducted at 30-min intervals, each structured with sequentially executed sample and choice phases. During the training sessions, the mice were immediately moved to the next stage after the prior phase ended.

During sample phase initiation, mice were positioned in the maze’s central stem with a barrier to block the starting point of the main trunk of the T-shaped maze. Following an adaptation period, the barrier was retracted vertically to permit the mice to pass through. Only one preselected target arm was opened; the other arm was closed off. After reward consumption, mice were returned to the starting point when the T maze was quickly cleaned and both arms were opened.

During the choice phase, mice were repositioned in the T-maze central stem, and during this choice run, mice were allowed to choose which of the 2 arms to visit. Reward pellets were exclusively provided in the arm opposite to the sample phase-selected path, and selecting the arm containing reward pellets was considered a correct choice. Upon entry into the previously sampled arm (error choice), immediate confinement was implemented and 30-s restricted mobility was maintained. If the accuracy rate reaches or exceeds 80% for 2 consecutive days (referred to as days to criterion), it is regarded as meeting the training standard and one can proceed to the formal test.

The testing sessions comprised 10 daily trials conducted at 30-min intervals, each structured with sequentially executed sample, delay, and choice phases. Upon completion of the sample phase, the mice return to the maze’s central stem, after which the barrier is closed. The barrier remains closed until it reopens, thereby allowing the mice to proceed to the next stage. This interval is designated as the delay phase.

Upon achieving criterion performance, mice were assessed over 2 consecutive 24-h cycles on their choice accuracy across 0-, 30-, 60-, and 90-s delay phases. Each delay was tested for 2 d with an equivalent number of trials per condition, and the final performance metrics were calculated by averaging the accuracy from these 2 d. For experiments that combined optogenetics, we focused solely on the 60-s delay paradigm.

### Optogenetic neuronal activity manipulation

Optogenetic modulation protocols were implemented with spectral specificity: Inhibitory halorhodopsin (NpHR) activation was achieved using a 570-nm laser (10 mW mm^−2^) terminated with microsecond precision at behavioral epoch offset. Optogenetic activation employed 473-nm blue light for ChR2 stimulation (DPSS laser, Inper Co. Ltd.) with precise irradiance control (0.1 to 5 mW/mm^2^). Surgical implantation procedures were conducted under pentobarbital sodium anesthesia (50 mg/kg, intraperitoneally). Optical fibers (200 μm diameter, Inper Co. Ltd.) were implanted into the mouse brains according to the coordinates (Nts neurons in the MM: ML: 0 mm, AP: −2.8 mm, DV: −5.7 mm; axons of Nts neurons in the AV: ML: ±0.98 mm, AP: −0.7 mm, DV: −3.1 mm; Nos1 neurons in the MM: ML: ±0.45 mm, AP: −2.8 mm, DV: −5.7 mm; axons of Nos1 neurons in the AV: ML: ±1.0 mm, AP: −0.7 mm, DV: −3.1 mm). Behavioral experiments were conducted following a 7-d postsurgical recovery period.

### Ca^2+^ photometry and data analysis

Mice underwent a 12-d habituation period to the experimenters and testing environments prior to any experimental manipulation. Two weeks after the rAAV2/9-hSyn-DIO-GCaMP6s was injected, an optical ceramic needle was inserted toward the MM through the craniotomy. All mice were maintained for a 7-d postsurgical convalescence period. Ca^2+^ activity in Nts^GCaMP6s^ or Nos1^GCaMP6s^ neurons was continuously monitored through fiber-based photometric recording (Inper Co. Ltd.) in freely moving mice. A 2-s pre-test recording was established as baseline reference prior to each test. Z-score was achieved through baseline-adjusted computation, involving mean subtraction of reference signals followed by standardization using baseline-derived variability metrics. A 488-nm laser (0.01 to 0.02 mW) was delivered using an optical fiber imaging system, and fluorescent signals were recorded during the DNMP T maze. Dual-wavelength excitation (465 nm for GCaMP6s, 405 nm for control) was delivered through a dichroic mirror during imaging sessions. Fiber output power was calibrated to 30 μW. In all tests, real-time signal processors (RX8 and RZ5P, Tucker-Davis Technologies) and software (OpenEx.v.2.20 for RX8 and Synapse v.90 for RZ5P, Tucker-Davis Technologies) were utilized to control the output of each light-emitting diode (LED) at a sampling rate of 50 Hz, thereby isolating the signals emitted by each LED. We established temporal alignment using a multimodal synchronization protocol where *t* = 0 corresponded to the maze entry initiation and barrier retraction, with pre-event baseline defined as the 15-s epoch preceding barrier movement. Mouse movements were tracked using automated video analysis supplemented by manual verification.

### Statistical analysis

Statistical analyses were performed using GraphPad Prism software (version 10.0; GraphPad Software Inc., La Jolla, CA, USA). Data are presented as the mean ± SEM. Unpaired 2-tailed Student’s *t* tests and Bonferroni’s post hoc tests were applied following 1-way or 2-way analysis of variance (ANOVA) when normality and equal variance assumptions were satisfied. Statistical significance was defined as *P* < 0.05. For all experimental procedures including viral quantification, Ca^2+^ imaging, and electrophysiological recordings, investigators were blinded to treatment conditions during data collection and analysis.

## Ethical Approval

All experimental procedures were conducted in strict compliance with the National Natural Science Foundation of China guidelines and received formal approval (ACUC number: HUST-IACUC-2025-0032) from the Institutional Animal Care and Use Committee at Huazhong University of Science and Technology, China.

## Data Availability

All data needed to evaluate the conclusions in the paper are present in the paper and/or the Supplementary Materials.
