# Supplementary material for: Two Molecularly Defined Neuronal Types in the Mammillary Body Govern Different Temporal Periods during Working Memory Maintenance
Source: Research (Wash D C). 2026 Apr 22;9:1253. doi: 10.34133/research.1253 (PMC13100351; doi:10.34133/research.1253)
Supplement: Supplementary 1 — Figs. S1 to S10 Tables S1 and S2 Movies S1 and S2 [file research.1253.f1.zip › Supplementary Figures and Tables.pdf]

Fig. S1. Elevated *Grik4* expression in Nos1 neurons.

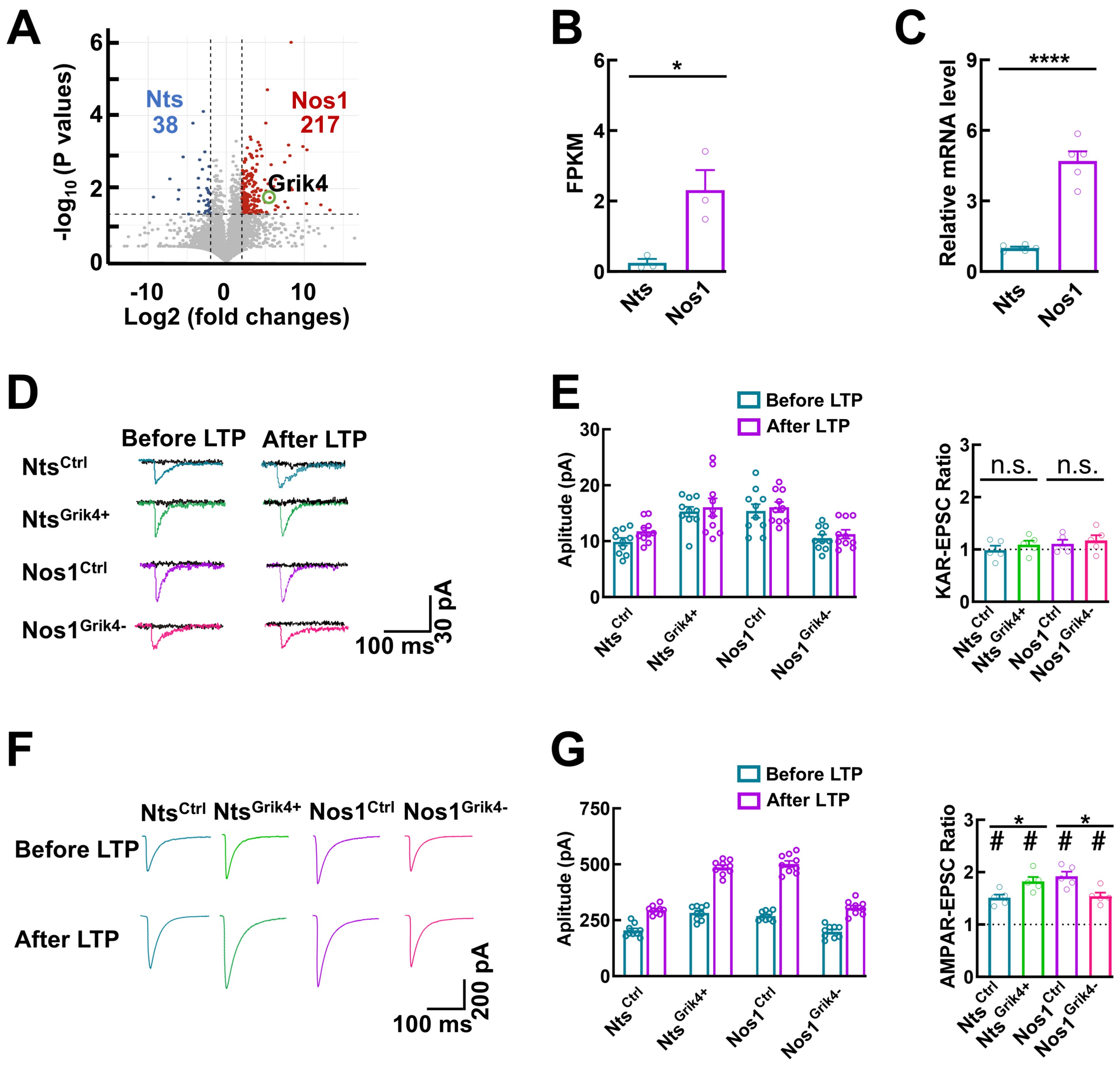

Fig. S1. Elevated *Grik4* expression in Nos1 neurons.

(A) A volcano plot showing the genes enriched in Nts (38genes, blue) versus Nos1 (217 genes, blue) neurons and both (grey) from the population cell mRNA-seq (n = 3 samples per group).

(**B**) A bar graph illustrating the gene levels of *Grik4* (mRNA-seq) in Nts and Nos1 neurons. Data are mean ± SEM (n = 3 per group, *P = 0.0243, t-test). FPKM: fragments per kilobase of transcript per million fragments mapped reads.

(**C**) A bar graph showing the mRNA level of *Grik4* (qPCR) in Nts and Nos1 neurons. Data are mean ± SEM (n = 5 per group, ****P < 0.0001, t-test).

(**D-E**) Representative traces showing KAR-mediated currents (**D**) and the ratios of KAR-mediated currents (**E**) from Nts^{Ctrl}, Nts^{Grik4+}, $Nos1^{Ctrl}$ and $Nos1^{Grik4-}$ neurons before and after LTP induction. To minimize potential confounders such as drug washout residual effects and inter-individual variability, we used separate slices from the same mice for pre- or post-LTP recordings. During the experiment, two brain slices were obtained from each mouse, and the amplitude of current of two neurons were recorded from each brain slice. In the data analysis, the mean value of the current differences within the same mouse was selected for comparison of the ratios before and after the LTP induction. Data are mean ± SEM (n = 10 neurons per group from 5 mice, t-test).

(**F-G**) Representative traces showing AMPAR-mediated currents (**F**) and the ratios of AMPAR-mediated currents (**G**) from Nts^{Ctrl}, Nts^{Grik4+}, $Nos1^{Ctrl}$ and $Nos1^{Grik4-}$ neurons before and after LTP induction. Data are mean ± SEM (n = 10 neurons per group from 5 mice, # indicates a within-group difference compared to the baseline (ratio=1), # P<0.05, t-test; * indicates a between-group difference, Nts^{Ctrl} versus Nts^{Grik4+} *P = 0.0177; $Nos1^{Ctrl}$ versus $Nos1^{Grik4-}$ *P = 0.0102, t-test).

Fig. S2. Excitatory projection from VS to Nts and Nos1 neurons.

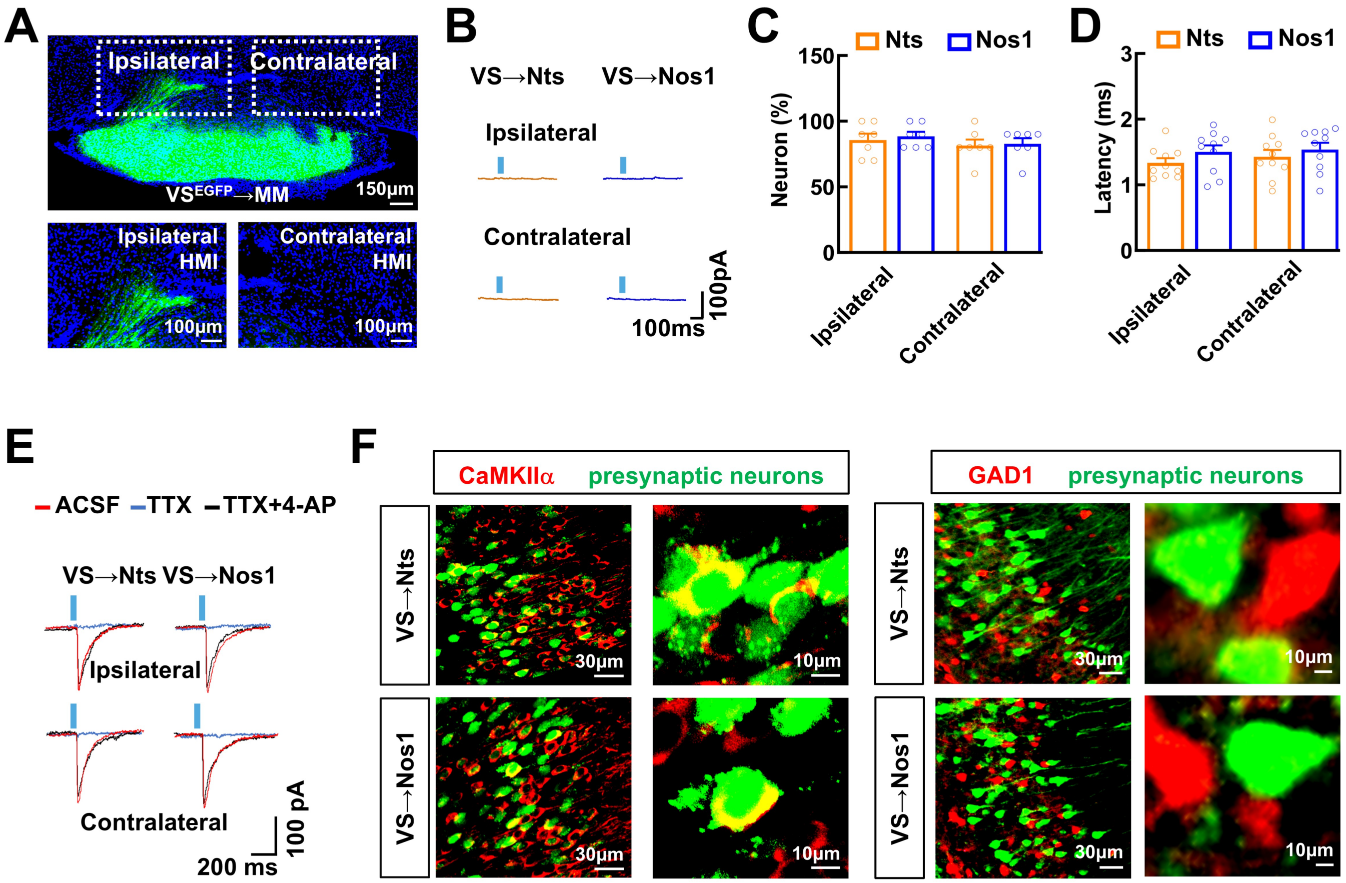

Fig. S2. Excitatory projection from VS to Nts and Nos1 neurons.

(A) Representative images (same brain section as in the upper panel of **Fig. 4D**) showing the axon from unilateral VS project into the MM with elevated exposure. Top: low-magnification image; Bottom: magnified view of the boxed region. HMI: high magnification image.

(B) Representative traces depicting no significant oIPSCs were recorded from bilateral Nts and Nos1 neurons (n = 10 neurons from 4 mice per group).

(C) A bar graph showing the proportion of neurons that exhibit EPSC responses under the given intensity of light stimulation. Data are mean ± SEM (n = 7 mice per group, with 10 neurons recorded in the MM on each side per mouse).

(D) A bar graph showing the latency of oEPSCs recorded from Nts and Nos1 neurons. Data are mean ± SEM (n = 10 neurons from 4 mice per group).

(E) Representative images showing the oEPSCs recorded from the NTS and Nos1 neurons were completely blocked by TTX and reversed by 4-AP.

(F) Representative images showing the presynaptic neurons (green) in VS of Nts and Nos1 neurons co-staining with excitatory (anti-CaMKIIα) and inhibitory (anti-GAD1) neuronal marker. HMI: high magnification image.

Fig. S3. Connectivity features from Nts and Nos1 neurons to the AV.

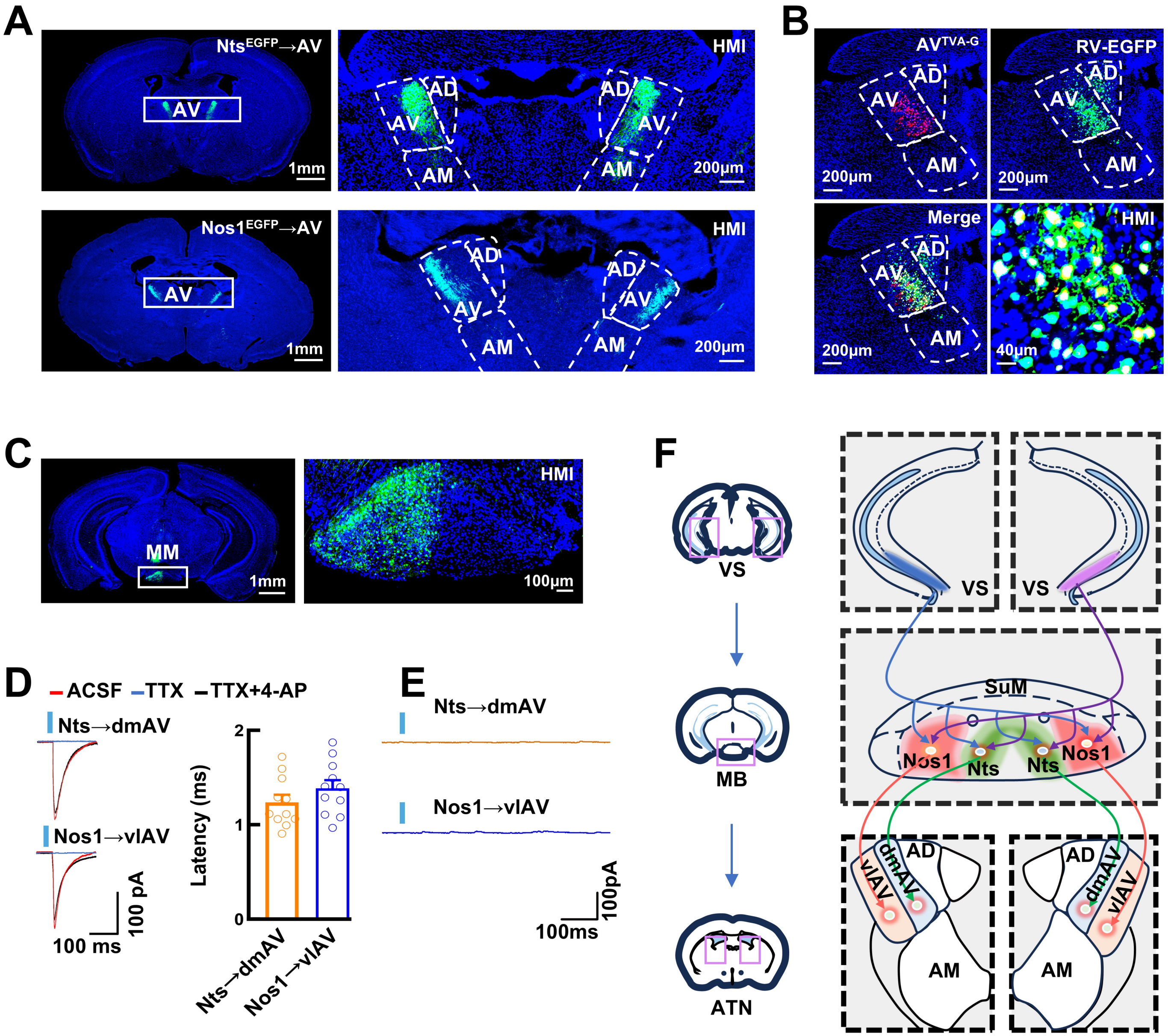

Fig. S3. Connectivity features from Nts and Nos1 neurons to the AV.

(A) Representative images illustrating the axon of bilateral Nts and Nos1 neurons (**Figure. 1D**) in the AV. HMI: high magnification image.

(B-C) Representative images showing EGFP in $AV^{TVA\text{-}G}$ neurons (**B**) and their presynaptic neurons in the MM (**C**) by injecting rAAV2/9-hSyn-DIO-mCherry-TVA-G and EnvA-RV(CVS-N2C)-ΔG-EGFP into the AV of wild-type mice . HMI: high magnification image.

(D) Representative images showing the oEPSCs recorded from the AV neurons were completely blocked by TTX and reversed by 4-AP. A bar graph showing the latency of oEPSCs recorded from the AV neurons. Data are mean ± SEM (n = 11 neurons from 4 mice per group).

(E) Representative traces depicting no significant oIPSCs were recorded in the AV neurons. (n = 11 neurons from 4 mice per group).

(F) Schematic illustrating the neural circuits of Nts and Nos1 neurons. Nts and Nos1 neurons in the MM receive excitatory inputs from VS with unilateral-to-bilateral pattern, and send the parallel excitatory projections to the ipsilateral dmAV and vlAV, respectively.

Fig. S4. Connectivity patterns between Nts and Nos1 neurons in the MM and the VTg.

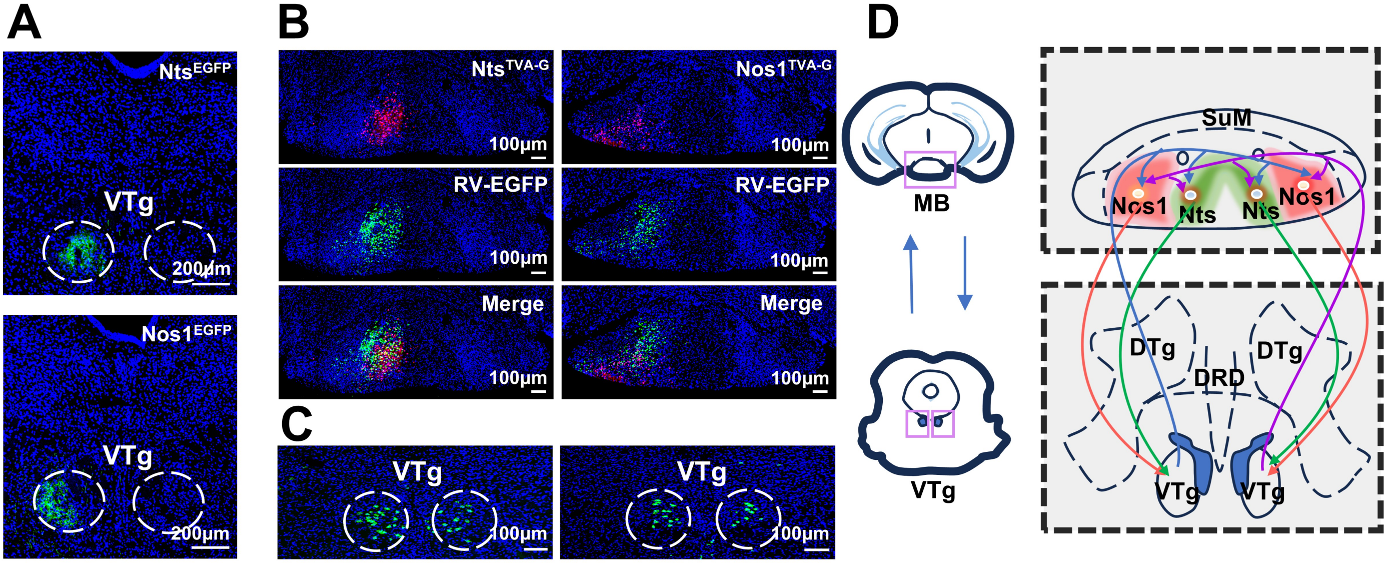

Fig. S4. Connectivity patterns between Nts and Nos1 neurons in the MM and the VTg.

(**A**) Representative images showing the EGFP-labeled terminal of unilateral Nts neurons and Nos1 neurons (**Fig. 5B**) in the VTg.

(B-C) Representative images showing EGFP in $Nts^{TVA\text{-}G}$ neurons and $Nos1^{TVA\text{-}G}$ neurons (**B**) and their presynaptic neurons (**C**) in the VTg by injecting rAAV2/2-retro-hSyn-DIO-Flpo in the AV and rAAV2/9-fDIO-mRuby3-TVA-G and EnvA-RV(CVS-N2C)-ΔG-EGFP into the MM of Nts^{CRE} and $Nos1^{CRE}$ mice.

(D) Schematic illustrating the precise connectivity patterns between Nts and Nos1 neurons and the VTg.

Fig. S5. Kir2.1 inhibits the neuronal activity.

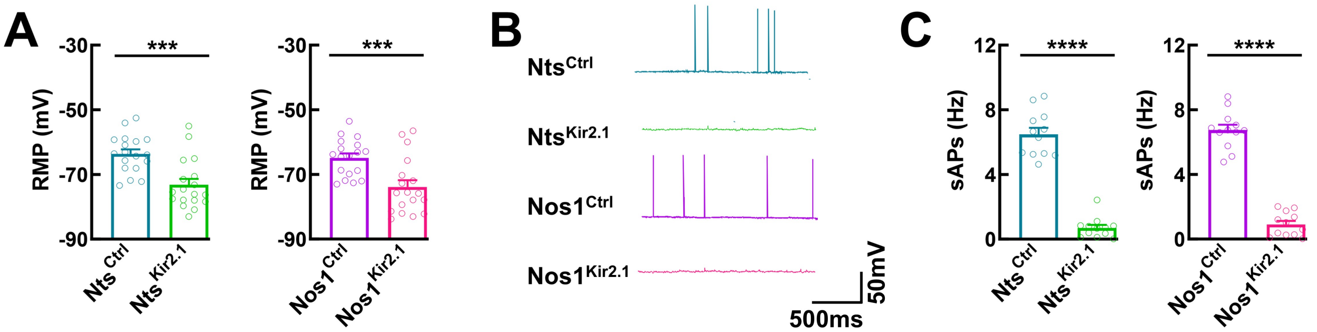

Fig. S5. Kir2.1 inhibits the neuronal activity.

(A) Bar graphs showing resting membrane potential (RMP) of Nts^{Ctrl}, $Nts^{Kir2.1}$, $Nos1^{Ctrl}$, and $Nos1^{Kir2.1}$ neurons. Data are mean ± SEM (n = 18 neurons from 6 mice per group, Nts^{Ctrl} versus $Nts^{Kir2.1}$ ***$P = 0.0002$; $Nos1^{Ctrl}$ versus $Nos1^{Kir2.1}$ ***$P = 0.009$, *t*-test).

(B-C) Representative traces and bar graphs showing spontaneous action potentials (sAPs) of Nts^{Ctrl}, $Nts^{Kir2.1}$, $Nos1^{Ctrl}$ and $Nos1^{Kir2.1}$ neurons. Data are mean ± SEM (n =12 neurons from 4 mice per group, Nts^{Ctrl} versus $Nts^{Kir2.1}$ ****$P < 0.001$; $Nos1^{Ctrl}$ versus $Nos1^{Kir2.1}$ ****$P < 0.001$, *t*-test).

Fig. S6. Nts^{Ctrl}, $Nts^{Kir2.1}$, $Nos1^{Ctrl}$ and $Nos1^{Kir2.1}$ mice underwent behavioral assessment.

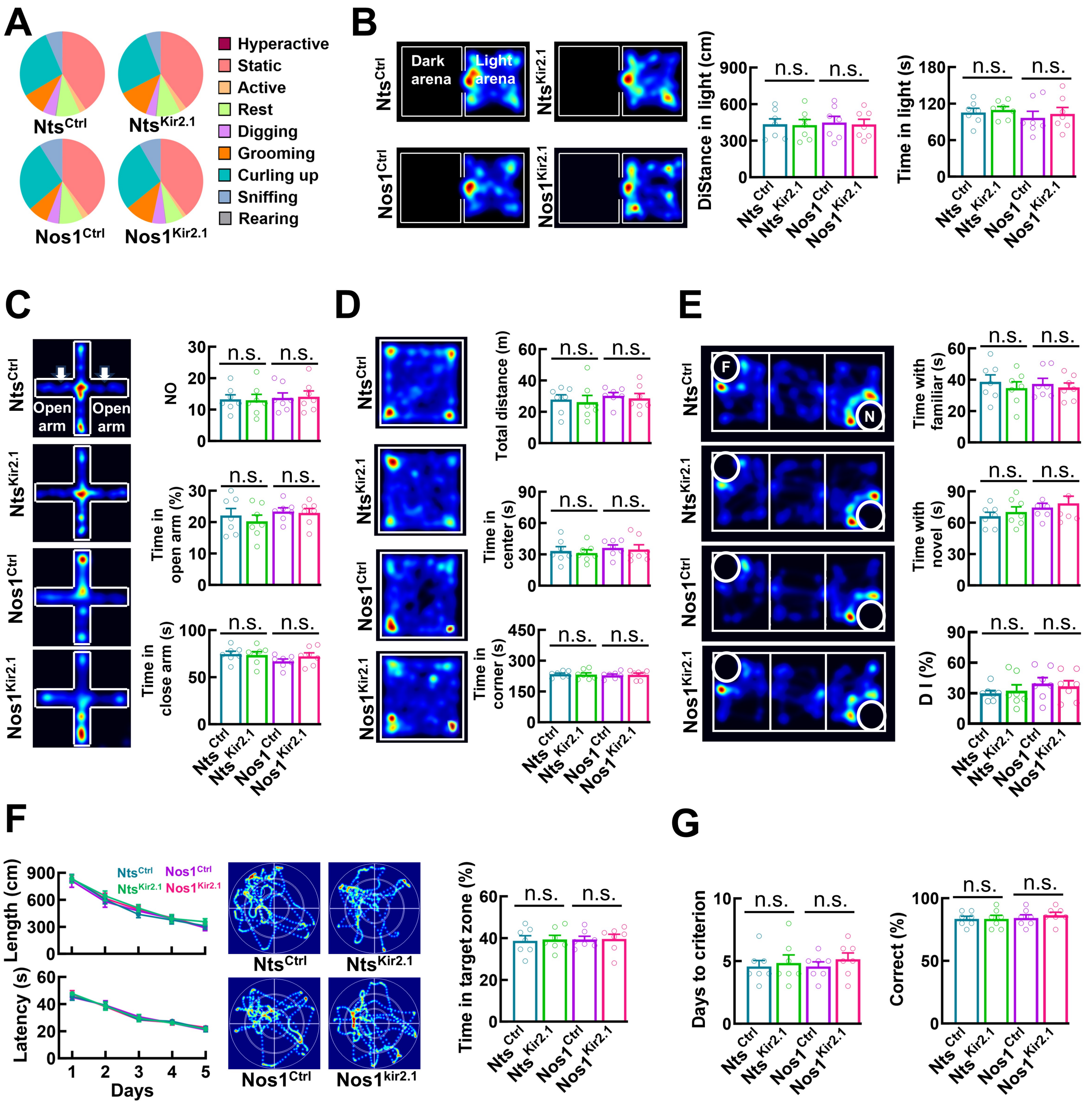

Fig. S6. Nts^{Ctrl}, Nts$^{Kir2.1}$, Nos1^{Ctrl} and Nos1$^{Kir2.1}$ mice underwent behavioral assessment.

(A) Pie chart analysis showing behavioral proportion in the home cage. Data are mean ± SEM (n = 7 mice per group).

(B) Heat maps and bar graphs illustrating the travel distance and time spent in the light arena of light-dark box. Data are mean ± SEM (n = 7 mice per group, *t*-test).

(C) Heat maps and bar graphs illustrating the number of entries into the open arm (NO) and time spent in the open ands closed arms of the elevated plus. Data are mean ± SEM (n = 7 mice per group, *t*-test).

(D) Heat maps and bar graphs illustrating the total travel distance and the time spent in the center and corner areas of open field. Data are mean ± SEM (n =7 mice per group, *t*-test).

(E) Heat maps and bar graphs illustrating the interaction time to familiar and novel individuals and the discrimination index (DI), in the three-chamber test. Data are mean ± SEM (n = 7 mice per group, *t*-test).

(F) Line graphs illustrating the latency and the swim length to reach a hidden platform during the training sessions in the Morris water maze. Heat maps and bar graph showing the percentage of time spent in searching of a hidden platform in targeting quadrant during the testing sessions in the Morris water maze. Data are mean ± SEM (n = 7 mice per group, two-way ANVOA for the line graphs and *t*-test for the bar graph).

(**G**) Bar graphs showing the training days to criterion and the performance in the testing sessions of DNMP T-maze within the 0s delay phases. Data are mean ± SEM (n = 7 mice per group, *t*-test).

Fig. S7. Nts→dmAV and Nos1→vlAV neural circuits govern different temporal periods during working memory maintenance.

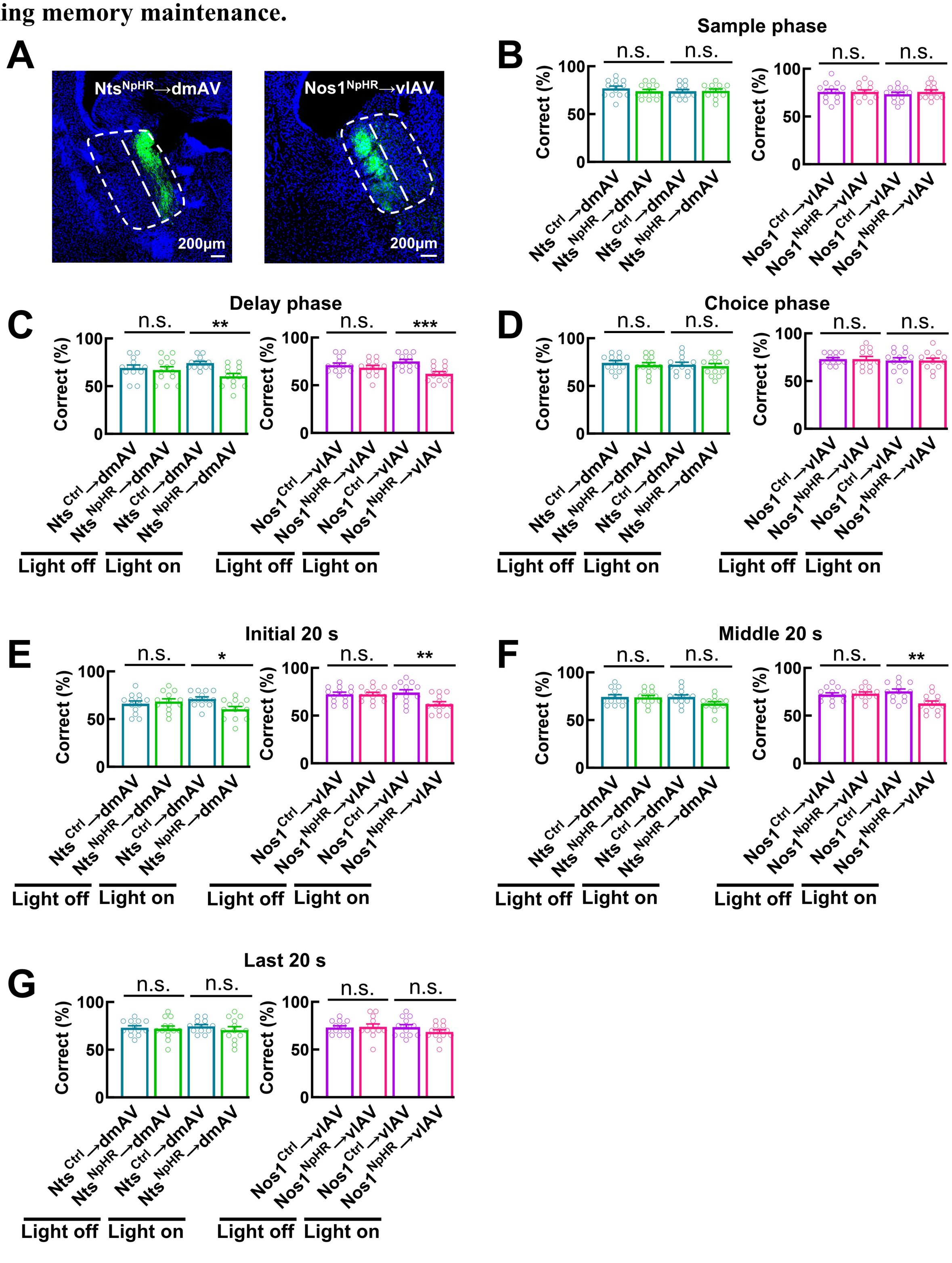

Fig. S7. Nts→dmAV and Nos1→vlAV neural circuits govern different temporal periods during working memory maintenance.

(**A**) Representative images showing the axon in the AV from Nts^{NpHR} and $Nos1^{NpHR}$ neurons.

(**B-D**) Bar graphs showing the performance of Nts^{Ctrl}, Nts^{NpHR}, $Nos1^{Ctrl}$ and $Nos1^{NpHR}$ mice in the testing sessions of DNMP T-maze with optical inhibition the during different phases. Optical inhibition was delivered to the axon by implanting the optical fiber to the AV. Data are mean ± SEM (n = 13 mice per group, Nts^{Ctrl} versus Nts^{NpHR} **P = 0.0086; $Nos1^{Ctrl}$ versus $Nos1^{NpHR}$ ***P = 0.0009, two-way ANOVA).

(**E-G**) Bar graphs showing the performance of Nts^{Ctrl}, Nts^{NpHR}, $Nos1^{Ctrl}$ and $Nos1^{NpHR}$ mice in the testing sessions of DNMP T-maze with optical inhibition the during the three 20 s periods within the 60 s delay phases. Optical inhibition was delivered to the axon by implanting the optical fiber to the AV. Data are mean ± SEM (n = 13 mice per group, Intial 20s Light on Nts^{Ctrl} versus Nts^{NpHR} *P = 0.0316, $Nos1^{Ctrl}$ versus $Nos1^{NpHR}$ **P = 0.0052; Middle 20s Light on $Nos1^{Ctrl}$ versus $Nos1^{NpHR}$ ** P = 0.0014, two-way ANOVA).

Fig. S8. Nts^{Ctrl}, Nts^{Grik4+}, Nos1^{Ctrl} and Nos1^{Grik4-} mice underwent behavioral assessment.

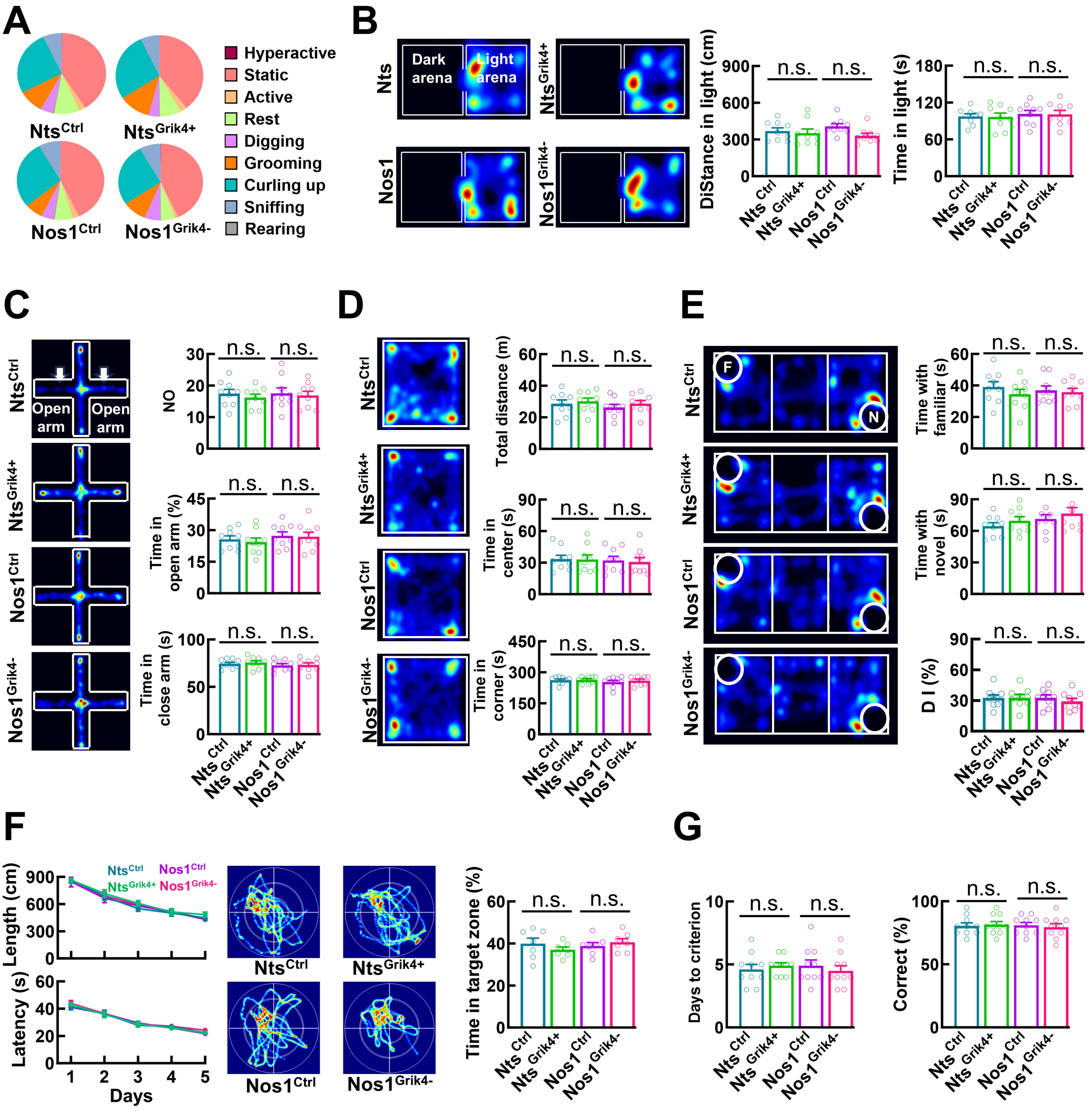

Fig. S8. Nts^{Ctrl}, Nts^{Grik4+}, $Nos1^{Ctrl}$ and $Nos1^{Grik4-}$ mice underwent behavioral assessment.

(A) Pie chart analysis showing behavioral proportion in the home cage. Data are mean ± SEM (n = 7 mice per group).

(B) Heat maps and bar graphs illustrating the travel distance and time spent in the light arena of light-dark box. Data are mean ± SEM (n = 9 mice per group, *t*-test).

(C) Heat maps and bar graphs illustrating the number of entries into the open arm (NO) and time spent in the open ands closed arms of the elevated plus. Data are mean ± SEM (n = 9 mice per group, *t*-test).

(D) Heat maps and bar graphs illustrating the total travel distance and the time spent in the center and corner areas of open field. Data are mean ± SEM (n =9 mice per group, *t*-test).

(E) Heat maps and bar graphs illustrating the interaction time to familiar and novel individuals and the discrimination index (DI), in the three-chamber test. Data are mean ± SEM (n = 9 mice per group, one-way ANOVA).

(F) Line graphs illustrating the latency and the swim length to reach a hidden platform during the training sessions in the Morris water maze. Data are mean ± SEM (n = 7 mice per group, two-way ANOVA); Heat maps and bar graph showing the percentage of time spent in searching of a hidden platform in targeting quadrant during the testing sessions in the Morris water maze. Data are mean ± SEM (n = 7 mice per group, two-way ANVOA for the line graphs and one-way ANOVA for the bar graph).

(**G**) Bar graphs showing the training days to criterion and the performance in the testing sessions of DNMP T-maze within the 0s delay phases. Data are mean ± SEM (n = 10 mice per group, one-way ANOVA).

Fig. S9. Temporal neuronal activity during working memory of Nts and Nos1 neurons.

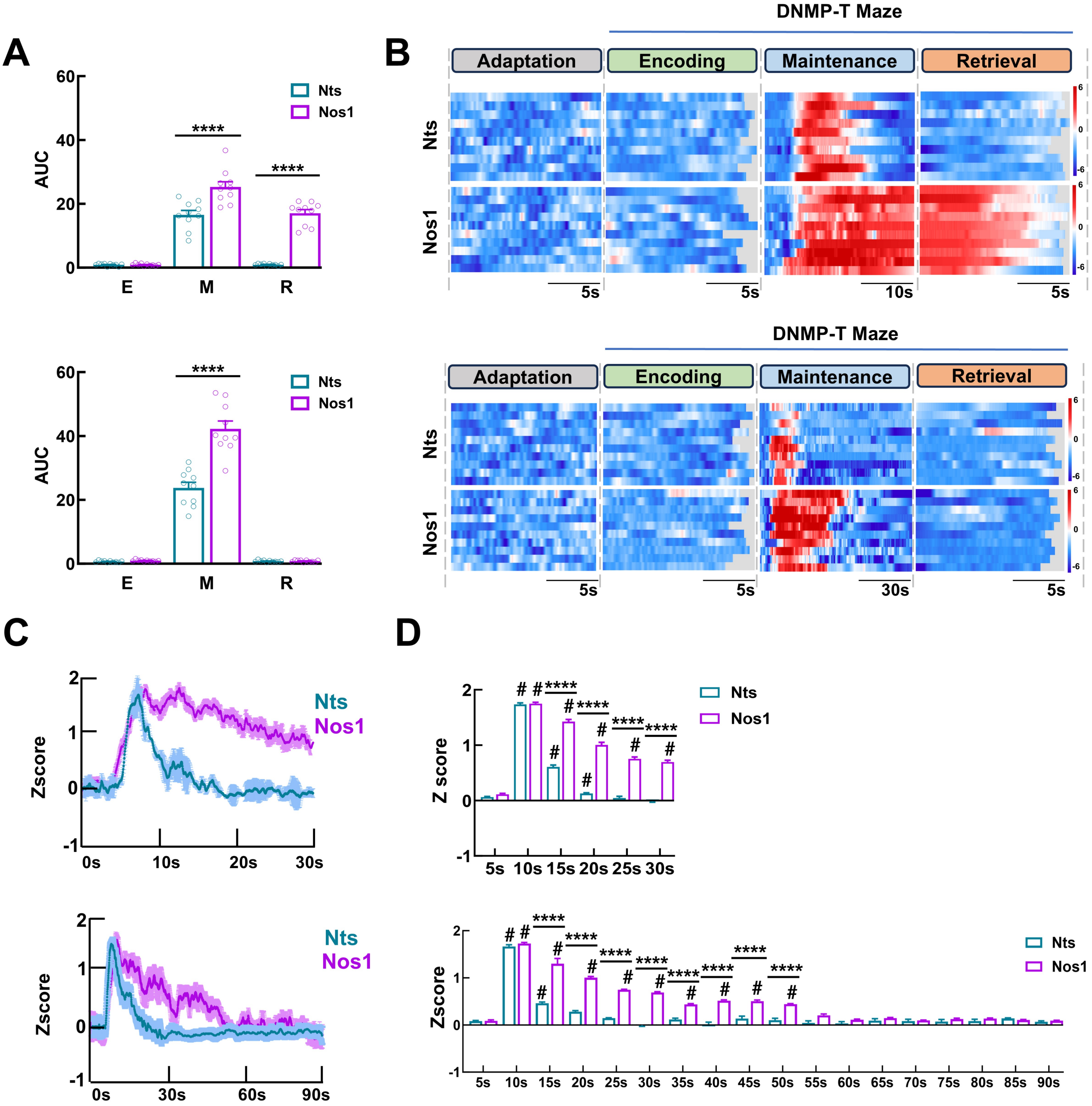

Fig. S9. Temporal neuronal activity during working memory of Nts and Nos1 neurons.

(**A-B**) Heat maps (**A**) and bar graphs (**B**) showing the neuronal activity of $Nts^{GCaMP6s}$ and $Nos1^{GCaMP6s}$ neurons during the encoding, maintenance (30 s and 90 s of delay phase) and retrieval of working memory of DNMP T-maze. Data are mean ± SEM (n = 10 mice per group, ****$P < 0.0001$, two-way ANOVA).

(C-D) Z-score of Ca^{2+} dynamics (**C**) and bar graphs (**D**, time binned at 5s) showing the temporal neuronal activity of $Nts^{GCaMP6s}$ and $Nos1^{GCaMP6s}$ neurons during the working memory maintenance (30 s and 90 s of delay phase) of DNMP T-maze. Data are mean ± SEM (n = 10 mice per group; # indicates a within-group difference compared to the baseline, #P<0.05, *t*-test; *indicates a between-group difference, ****P<0.0001, two-way ANOVA).

Fig. S10. Two molecularly-defined neuronal types in the mammillary body govern different temporal periods during working memory maintenance.

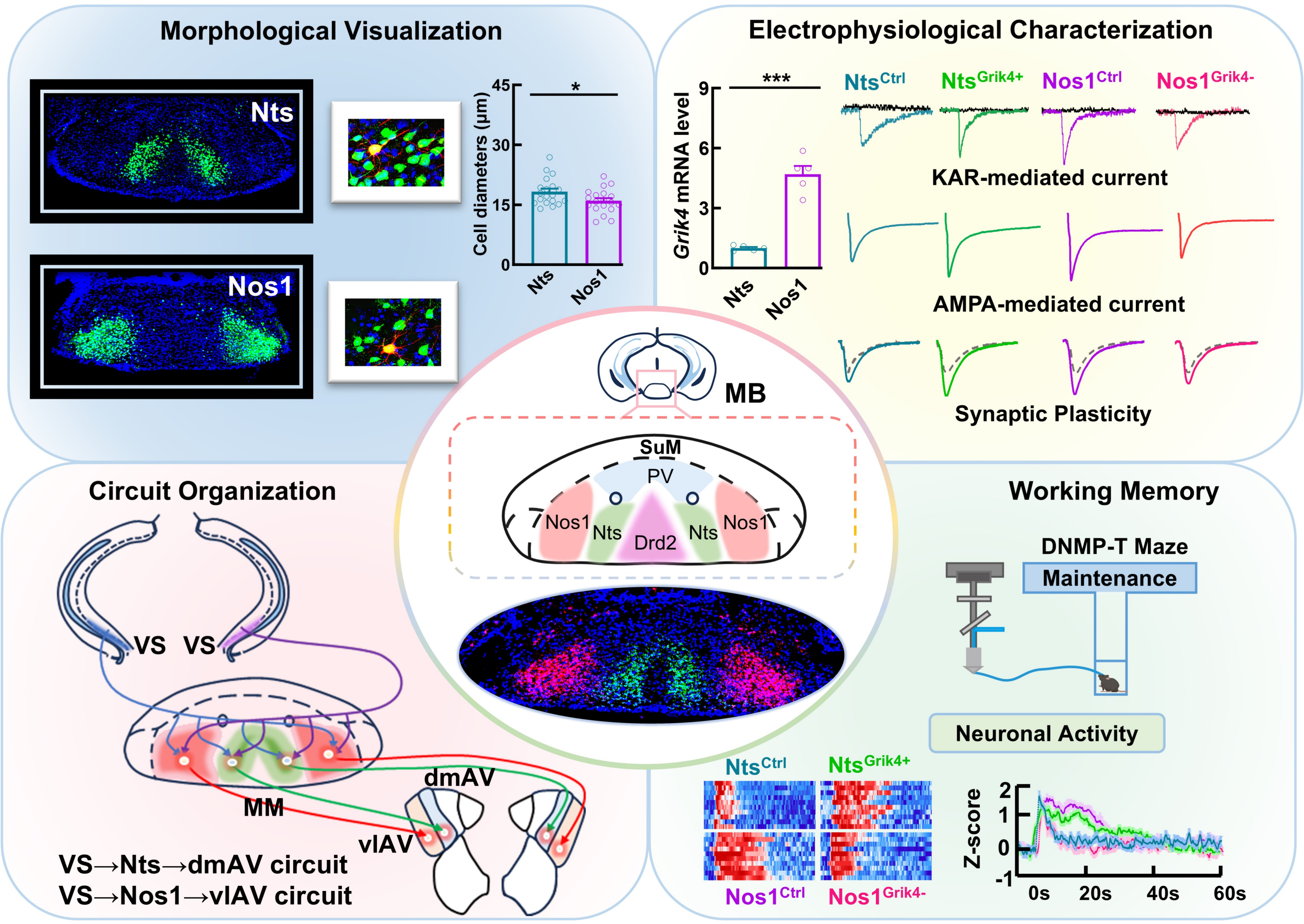

Fig. S10. Two molecularly-defined neuronal types in the mammillary body govern different temporal periods during working memory maintenance.

Nts and Nos1 neurons were organized into bilaterally symmetrical subgroups within the lateral regions of the MM. *Grik4* mediates differential synaptic transmission between Nts and Nos1 neurons. Nts and Nos1 neurons receive unilateral-to-bilateral inputs from the VS and ipsilaterally project in parallel to the dmAV and vlAV. Both Nts and Nos1 neurons are essential for working memory maintenance but contribute to distinct temporal phases of this process.

Table S1. Enriched genes in Nts neurons

Gene_name	Gene_ID	Nos1-1 FPKM	Nos1-2 FPKM	Nos1-3 FPKM	Nts-1 FPKM	Nts-2 FPKM	Nts-3 FPKM	log2F.C.	P value
Astn1	ENSMUSG00000026587	18.762163	12.699256	19.357714	32.341698	45.512768	27.552275	-1.05252	0.0345
Ktn1	ENSMUSG00000021843	17.08106	22.593201	10.012272	32.567574	35.416916	28.013395	-0.95015	0.02181
Mmd	ENSMUSG00000003948	30.556318	11.82579	6.061352	40.046516	35.370392	43.953827	-1.30108	0.03873
Pura	ENSMUSG00000043991	18.779989	9.272274	20.093399	41.02903	67.427811	33.394485	-1.5589	0.04518
Stam	ENSMUSG00000026718	21.61829	13.339351	13.153817	34.905521	21.896946	33.198559	-0.90356	0.04764
Dzank1	ENSMUSG00000037259	13.445009	15.289794	18.799538	21.129538	22.190485	19.25606	-0.39664	0.04872
Sec23a	ENSMUSG00000020986	18.199284	13.047004	14.912001	35.460171	51.061859	31.494118	-1.35432	0.01771
Ap3d1	ENSMUSG00000020198	15.958669	16.311838	11.195219	30.606977	37.304623	28.812216	-1.15399	0.00442
Ctnnbip1	ENSMUSG00000028988	14.215563	10.68089	14.69594	33.038212	43.080559	26.130274	-1.36879	0.01473
Smc3	ENSMUSG00000024974	13.152964	18.724459	6.57745	26.930727	25.634644	20.696638	-0.9299	0.04381
Cryab	ENSMUSG00000032060	25.2773	12.659282	0	84.438133	91.552757	77.681953	-2.74131	0.00099
Ccdc88a	ENSMUSG00000032740	14.619165	15.561503	6.929786	24.324701	19.859711	19.284361	-0.77422	0.04998
Rhob	ENSMUSG00000054364	13.077432	8.950957	14.74796	20.321217	31.123951	19.639545	-0.95076	0.04932
Gspt1	ENSMUSG00000062203	15.088117	10.574615	8.571094	18.257021	21.621292	21.18524	-0.83489	0.01523
Tmf1	ENSMUSG00000030059	14.196631	10.085548	9.357859	20.862627	50.130257	38.471848	-1.70221	0.043
Anp32a	ENSMUSG00000032249	7.952013	1.67592	23.448957	31.212036	37.954487	63.290253	-2.00163	0.04745
Ccdc184	ENSMUSG00000029875	11.599177	13.224352	7.983976	44.277405	29.699749	20.454935	-1.52525	0.04451
Dpp3	ENSMUSG00000063904	11.967156	12.538732	6.797822	17.752155	24.45973	17.457502	-0.93066	0.03193
Lhx1os	ENSMUSG00000087211	0.223037	0	7.598164	100.224663	186.886978	267.181915	-6.14712	0.01955
Nts	ENSMUSG00000019890	16.946117	0	0.647947	461.380463	119.993294	30.600302	-5.12031	0.02875
Zng1	ENSMUSG00000024878	0	11.626299	12.251927	28.019753	46.808346	35.298611	-2.2054	0.01317
Nrp1	ENSMUSG00000025810	4.119173	11.534816	6.103157	22.644201	41.122288	39.097126	-2.24117	0.01244
Kiz	ENSMUSG00000074749	2.546098	2.201985	2.097803	17.7882	21.901037	39.406273	-3.53029	0.0221
Adgrb1	ENSMUSG00000034730	16.365364	3.234472	5.222269	25.642471	25.917236	26.544189	-1.65377	0.01228
Efr3b	ENSMUSG00000020658	7.99575	7.196085	4.964323	15.204636	30.770424	28.403301	-1.88366	0.02142
Adam10	ENSMUSG00000054693	2.623554	5.311987	11.729179	20.663729	18.435865	33.195698	-1.87829	0.03016
Bmal1	ENSMUSG00000055116	2.918991	0.655597	3.997778	14.990711	16.86655	8.003022	-2.39614	0.01997
Atn1	ENSMUSG00000004263	4.319157	3.20784	3.740314	10.943895	17.716864	11.136246	-1.82052	0.01339
Paqr4	ENSMUSG00000023909	0.075252	4.91277	4.343406	13.807705	7.998302	17.951385	-2.09105	0.03601
Amotl1	ENSMUSG00000013076	7.506444	10.271816	9.22558	13.459464	14.066042	11.981879	-0.54896	0.01484
Map3k2	ENSMUSG00000004085	3.234328	1.463187	0.269683	0.002653	0.002038	0	-2.93495	7.8E-05
Slc24a2	ENSMUSG00000037996	0.498775	1.131541	4.536143	4.963049	7.18385	8.783587	-1.73151	0.02073
Zfp935	ENSMUSG00000113450	0.100181	4.926352	5.51472	6.957096	14.058273	13.162444	-1.69701	0.04889
Nr1d1	ENSMUSG00000020889	2.789057	4.651716	5.340519	12.578873	6.730942	14.022125	-1.38287	0.04377
Amd2	ENSMUSG00000063953	4.978892	5.331292	9.320551	10.938403	9.703072	11.641069	-0.71764	0.04852
Pygo2	ENSMUSG00000047824	1.278208	2.325041	6.57955	12.692418	12.509655	6.763435	-1.65038	0.04564
Nup133	ENSMUSG00000039509	0.497731	3.878491	1.320432	13.25843	11.859981	5.816588	-2.44105	0.02817
Tenm3	ENSMUSG00000048661	0.461086	4.186212	3.755476	12.706931	6.38372	11.841637	-1.88018	0.03104

Table S2. Enriched genes in Nos1 neurons									
Gene_name	Gene_ID	Nos1-1 FPKM	Nos1-2 FPKM	Nos1-3 FPKM	Nts-1 FPKM	Nts-2 FPKM	Nts-3 FPKM	log2F.C.	P value
Nnat	ENSMUSG00000067786	719.652222	719.833923	563.7052	328.367798	166.657547	98.458923	0.0054	0.0054
Eno1	ENSMUSG00000063524	272.387421	413.301575	520.513611	221.148468	129.812653	203.109344	1.12233	0.04783
Atraid	ENSMUSG00000013622	281.618958	254.214294	181.283478	135.224152	29.73105	106.261078	1.40276	0.02674
Dctn3	ENSMUSG00000028447	240.936722	194.970169	277.142059	95.545517	92.442177	151.932205	1.06881	0.01537
Eno2	ENSMUSG00000004267	172.097549	222.003235	187.731476	118.321617	64.463783	59.421848	1.26436	0.00909
Eif1b	ENSMUSG00000006941	219.524094	148.169846	169.08609	114.966995	50.977634	96.228157	1.03381	0.03235
Matk	ENSMUSG00000004933	242.004257	110.902756	164.295105	48.409439	3.792565	60.058655	2.20388	0.03187
Aplp2	ENSMUSG00000031996	116.789261	188.112518	191.307373	67.382553	113.498466	64.479149	1.01605	0.04508
Ssbp4	ENSMUSG00000070003	157.868423	153.906662	160.671326	63.07933	28.46707	109.592094	1.23196	0.01855
Gde1	ENSMUSG00000033917	135.100113	156.07811	175.362869	103.929405	53.302212	26.105064	1.34751	0.02105
Eif3g	ENSMUSG00000070319	107.451614	125.756287	181.646332	78.493797	0	59.676888	1.58615	0.04707
Afdn	ENSMUSG00000068036	190.802078	119.452187	84.325417	23.726093	42.60461	43.859566	1.84032	0.04143
Cmas	ENSMUSG00000030282	141.737885	159.292297	91.286659	47.894756	37.652626	48.300404	1.55143	0.01407
Ppp1ca	ENSMUSG00000040385	63.150612	121.332031	173.665161	18.956039	6.816015	30.444889	2.67148	0.0368
Pdlim7	ENSMUSG00000021493	103.265625	130.142715	122.680222	18.584509	6.642822	49.861942	2.24556	0.0035
Jpt1	ENSMUSG00000020737	140.8405	106.621895	103.239395	72.521469	0.213195	11.432096	2.05892	0.02518
Camk1	ENSMUSG00000030272	104.40596	145.189285	80.826096	34.248016	3.368676	52.829899	1.86917	0.0279
Mrps34	ENSMUSG00000038880	116.429893	103.577286	107.886955	72.064224	1.910444	51.612358	1.38454	0.03336
Mrps17	ENSMUSG00000034211	137.931564	115.509727	70.27475	31.618883	0.168993	0.715418	3.31607	0.01241
Adissp	ENSMUSG00000027327	69.524673	107.124969	109.920601	57.724934	13.673462	20.374538	1.64275	0.02643
Gsta4	ENSMUSG00000032348	82.37603	130.548508	72.028946	49.787933	0.184529	0	2.51152	0.03293
Lsm4	ENSMUSG00000031848	119.308975	58.858734	105.813004	41.688972	0	26.428787	2.05969	0.03076
Ndufv1	ENSMUSG00000037916	100.775177	84.432594	96.366524	20.040579	37.093273	65.69281	1.19689	0.02029
Pcyt2	ENSMUSG00000025137	74.522331	111.337006	81.746109	38.098141	27.470596	3.230187	1.95965	0.01227
Prepl	ENSMUSG00000024127	79.018089	102.274162	58.757599	32.282288	9.140114	38.826424	1.58078	0.02619
Snx6	ENSMUSG00000005656	70.175743	69.703598	88.129982	24.340105	33.799446	0	1.9715	0.00854
Sec61a2	ENSMUSG00000025816	86.818253	94.495346	39.535076	33.801113	16.338438	0	2.13904	0.04504
Mrps26	ENSMUSG00000037740	54.298035	83.082321	71.72731	6.937923	19.330437	8.552733	2.58621	0.00326
Rapgef4	ENSMUSG00000049044	51.000698	100.117928	55.887379	29.996208	3.73661	2.8937	2.49871	0.03421
Tomm40	ENSMUSG00000002984	39.76136	86.299355	69.693542	11.283378	23.721033	31.575655	1.55588	0.04408
Rp9	ENSMUSG00000032239	70.921516	58.45219	58.784275	10.988345	29.350801	9.199071	1.92533	0.00376
Apmap	ENSMUSG00000033096	33.816929	75.252739	64.807899	16.507675	0	28.685478	1.9439	0.04562
Shisa5	ENSMUSG00000025647	56.395638	73.660217	43.360947	16.796892	5.235708	0	2.97653	0.00746
Pabir1	ENSMUSG00000074922	71.134453	49.99987	50.243286	20.633749	26.847498	0	1.85175	0.01826
Smarca2	ENSMUSG00000024921	41.863022	73.609421	53.244541	30.712021	22.902367	13.390738	1.33226	0.03248
Smarcb1	ENSMUSG00000000902	64.077927	71.019043	32.843655	7.581848	0	0.582962	4.36239	0.01132
Gemin7	ENSMUSG00000044709	49.113327	61.282768	53.906338	32.856026	0	24.441452	1.51981	0.02711
Poldip2	ENSMUSG00000001100	50.667568	38.201916	73.679153	21.493149	18.137924	9.42368	1.72841	0.02628
Rbfox1	ENSMUSG00000008658	53.887875	46.301971	61.245712	23.907944	7.468555	32.256283	1.34312	0.01827
Cyp46a1	ENSMUSG00000021259	47.400085	61.451859	48.107632	2.971369	38.143238	8.3415	1.66617	0.03907
Stk16	ENSMUSG00000026201	41.556435	70.039932	44.462116	14.851495	2.590776	22.288425	1.97376	0.0224
Mydgf	ENSMUSG00000019579	45.438404	67.795509	40.504299	22.188393	7.676496	22.196125	1.5622	0.02496
Micu1	ENSMUSG00000020111	58.007988	46.627422	47.342861	1.013011	4.255293	31.482117	2.04803	0.02056
Marchf2	ENSMUSG00000079557	70.87632	49.366467	30.487217	9.16273	0.104977	3.579335	3.55246	0.01842
Gramd1a	ENSMUSG00000001248	50.117393	62.336243	36.16827	3.822273	18.445223	24.297474	1.67433	0.02479
Clptm1l	ENSMUSG00000021610	34.92807	60.727413	52.813675	7.221869	3.188509	21.327486	2.22589	0.01441
Grik4	ENSMUSG00000032017	2.025079	3.412996	1.18546	0.464421	0.152845	0.12432	1.44778	0.04085
Wdr54	ENSMUSG00000030032	28.500767	45.317822	59.901821	5.10607	6.280202	0.473782	3.49504	0.01174
Tpra1	ENSMUSG00000002871	51.346447	58.947399	23.134367	10.372037	0.943459	10.489635	2.61332	0.0305
Tmub2	ENSMUSG00000034757	38.596146	59.476151	35.322834	0.838974	6.1189	20.214287	2.2955	0.0205
Taf11	ENSMUSG00000024218	67.052414	46.300652	19.847794	0.022048	11.166239	1.912264	3.3459	0.04678
Rhbdd2	ENSMUSG00000039917	28.881792	52.181263	51.861183	23.02162	19.080915	16.482649	1.182	0.03556
Neu1	ENSMUSG00000007038	48.790718	44.708969	37.926334	10.257745	5.980915	23.509344	1.7253	0.00768
Ubac1	ENSMUSG00000036352	51.74551	51.102222	27.903997	17.32567	0	21.649082	1.74622	0.04066
Ubald1	ENSMUSG00000039568	28.79884	40.140858	58.30994	23.51004	1.868273	7.607719	1.94773	0.04323
Dgcr6	ENSMUSG00000003531	48.926945	35.108727	42.645573	20.683907	0.98278	9.375316	2.02891	0.01021
Mterf3	ENSMUSG00000021519	43.143318	55.92218	27.230991	4.735833	10.244798	21.191334	1.80387	0.03526
Dek	ENSMUSG00000021377	38.652004	43.556946	44.003189	13.095359	0	29.643623	1.56223	0.03349
Tyw5	ENSMUSG00000048495	52.995728	37.687973	35.00066	26.568605	6.394347	14.79493	1.39599	0.03271
Shfl	ENSMUSG00000038884	30.218361	45.321514	46.482544	12.259213	6.425843	17.500729	1.75365	0.00958
Bbs4	ENSMUSG00000025235	47.647942	30.980738	43.241787	0.137608	0	0.292129	8.14768	0.00125
Arf2	ENSMUSG00000062421	25.859133	35.56583	58.879227	6.368404	10.866526	3.188049	2.55842	0.02956
Arc	ENSMUSG00000022602	37.152096	23.004303	59.652912	11.272506	2.648492	6.148115	2.57769	0.03866
Blmh	ENSMUSG00000020840	30.146673	51.840961	34.177849	15.279919	9.927071	6.496016	1.87349	0.01688
Usp33	ENSMUSG00000025437	35.722668	42.326031	37.54166	15.821797	0.490191	0.001323	2.8249	0.00398
Tsfm	ENSMUSG00000040521	46.752029	23.006359	40.845745	5.450575	0	18.176731	2.22688	0.0316
Ctsz	ENSMUSG00000016256	40.82193	40.660595	29.034529	0.023815	0.098788	0	9.81606	0.00071
Gars	ENSMUSG00000029777	28.864311	35.899544	45.473198	20.989782	3.771881	12.670388	1.55826	0.02473
Ppp1r35	ENSMUSG00000029725	41.113804	27.973608	40.915657	24.387159	4.972405	11.347374	1.4342	0.03236
Med4	ENSMUSG00000022109	40.38876	50.131405	15.955003	0.106339	0	12.894315	3.03386	0.04759
Ccdc74a	ENSMUSG00000041617	43.512974	28.02219	34.786327	24.562199	0	0	2.11392	0.04326
Dnaja3	ENSMUSG00000004069	41.669357	39.348774	24.76033	12.702227	9.662298	18.793362	1.36181	0.02216
Eif3l	ENSMUSG00000033047	24.243008	51.621746	29.124718	8.837091	9.403282	4.343648	2.21687	0.03288
Ddx1	ENSMUSG00000037149	22.522919	32.774158	45.601788	11.825573	11.09323	9.30035	1.64692	0.02709
Nup88	ENSMUSG00000040667	24.952026	38.140316	34.567829	10.990578	10.595983	6.01905	1.82281	0.00534
Mfsd11	ENSMUSG00000020818	35.226578	30.028284	31.896305	10.244038	0	7.55075	2.44878	0.00151

Lypla2	ENSMUSG00000028670	33.924328	36.313469	24.26549	3.437737	20.118084	0	2.00428	0.03066
Trmt2a	ENSMUSG00000022721	23.856289	34.358555	34.726566	7.762653	0.719798	0.693334	3.34042	0.00283
Car10	ENSMUSG00000056158	40.913597	30.983995	21.002832	13.022565	9.300505	7.029861	1.66218	0.02432
Pgam1-ps2	ENSMUSG00000082016	25.790884	35.452583	31.11338	20.235476	6.418462	16.415821	1.10054	0.02991
Elp2	ENSMUSG00000024271	37.777485	31.919268	22.648964	11.879544	14.016577	13.092259	1.244	0.01614
Zfp948	ENSMUSG00000067931	15.304173	38.419857	37.898251	10.170024	0	8.241262	2.31511	0.04139
Wdr70	ENSMUSG00000039828	43.921417	17.079567	29.009426	5.713515	6.814397	7.691585	2.15434	0.04042
Sin3b	ENSMUSG00000031622	38.43539	20.334606	30.942293	9.262559	5.759306	3.248809	2.29578	0.01261
Znhit2	ENSMUSG00000075227	23.432669	26.365213	38.653526	8.23539	12.13256	0	2.11858	0.01811
Wars1	ENSMUSG00000021266	31.595573	43.717812	13.052773	7.210077	1.163751	1.756064	3.12488	0.04598
Mapk1ip1	ENSMUSG00000041775	24.811012	23.440121	38.971764	6.738085	5.446331	6.78245	2.20123	0.0103
Tmem25	ENSMUSG00000002032	24.776642	31.466364	30.817352	15.62295	6.999704	4.306272	1.69286	0.00759
Stx1a	ENSMUSG00000007207	30.476227	27.405697	28.153471	10.470728	0.081346	6.541275	2.3315	0.00191
Tmem176b	ENSMUSG00000029810	31.421673	38.400387	15.892566	0	0.139846	11.884336	2.8336	0.03361
Grn	ENSMUSG00000034708	33.783047	29.362644	22.001633	7.676924	5.678221	0	2.67257	0.00443
Gm4735	ENSMUSG00000064193	21.294525	27.625988	35.980438	12.88789	5.721867	11.348833	1.50281	0.01857
Fndc3a	ENSMUSG00000033487	28.510246	27.767084	28.487799	15.967642	1.158243	18.405775	1.25437	0.03832
Sec61a1	ENSMUSG00000030082	31.750082	18.806019	34.157593	2.32076	4.885897	0	3.55519	0.00653
Spock1	ENSMUSG00000056222	16.905552	30.555208	37.024132	12.926191	5.357324	2.640107	2.01356	0.03381
Sf3b3	ENSMUSG00000033732	31.74876	29.500582	21.983681	5.246559	11.605887	18.126614	1.25066	0.02759
Med8	ENSMUSG00000006392	23.304127	32.713547	27.210768	7.138549	2.951655	9.545619	2.08359	0.00316
Gnl1	ENSMUSG00000024429	17.36187	34.919907	28.896154	9.653224	2.087474	4.02667	2.36415	0.01794
Ppp2r2a	ENSMUSG00000022052	17.421024	30.464167	32.694176	9.766983	0.545052	8.852243	2.07199	0.02159
Pcbp3	ENSMUSG00000001120	20.600714	29.269032	30.614313	0	1.698014	1.689719	4.57031	0.00129
Commd8	ENSMUSG00000029213	28.065384	24.682554	26.981133	15.617278	2.896977	14.22447	1.28411	0.01953
Rasal2	ENSMUSG00000070565	37.10223	20.199619	22.126503	2.611788	5.456588	10.020824	2.13453	0.02382
Fyco1	ENSMUSG00000025241	15.418716	42.240257	21.538202	6.515459	0	1.98761	3.21939	0.04758
Mrps9	ENSMUSG00000060679	23.688665	36.905125	18.579878	13.565455	0	0	2.54508	0.03676
Gns	ENSMUSG00000034707	22.330585	27.548223	28.680128	18.177908	3.772817	4.246155	1.58438	0.02694
Gna11	ENSMUSG00000034781	34.387535	21.50746	21.98641	2.423583	2.873514	10.254671	2.3242	0.01346
Ahcyl1	ENSMUSG00000027893	25.427898	29.772665	22.322742	6.790464	10.021362	15.701385	1.2536	0.01139
Camkmt	ENSMUSG00000071037	37.177105	25.627918	11.510284	0	0	6.00098	3.63039	0.04145
Madd	ENSMUSG00000040687	23.829859	15.361248	35.113636	0.85329	5.572478	11.894701	2.02	0.04654
Glb1	ENSMUSG00000045594	16.95693	27.34255	29.888941	10.26814	0	3.825281	2.39617	0.01564
Cacfd1	ENSMUSG00000015488	19.662483	20.956028	33.374611	2.503305	9.372208	1.002383	2.52249	0.01593
Rbck1	ENSMUSG00000027466	27.985577	20.28187	25.259834	7.415534	7.846387	0.727213	2.20119	0.00401
Hrh3	ENSMUSG00000039059	19.081045	21.50647	31.388998	3.562547	0	4.109942	3.22976	0.00574
Fam114a2	ENSMUSG00000020523	20.009808	20.53117	31.254875	5.270388	4.83647	6.81683	2.08486	0.0079
Hrh3	ENSMUSG00000039059	29.842705	23.511	16.607756	4.437932	0	11.340283	2.14863	0.02323
Uckl1	ENSMUSG00000089917	24.865778	21.977333	22.586843	14.509402	2.228308	0	2.05246	0.01875
Thumpd1	ENSMUSG00000030942	18.404013	17.225861	33.669712	2.995613	7.354309	0.987712	2.61173	0.02634
Cul2	ENSMUSG00000024231	16.166265	27.718473	25.244379	7.085336	10.544489	11.954066	1.22448	0.02557
Wdr45	ENSMUSG00000039382	21.346222	24.15897	23.621267	0	4.814027	12.215913	2.02116	0.00897
Zmym1	ENSMUSG00000043872	18.368029	27.326548	22.85956	1.739805	9.797607	0	2.57092	0.00876
Atpaf2	ENSMUSG00000042709	20.144703	25.067366	19.306091	7.856593	7.483453	0	2.0724	0.00633
Mvb12a	ENSMUSG00000042709	20.144703	25.067366	19.306091	7.856593	7.483453	0	4.17389	0.01579
Rgs9	ENSMUSG00000020599	23.057604	30.47608	10.105924	5.02038	3.895554	3.711838	2.33332	0.0464
Ubr3	ENSMUSG00000044308	19.597254	20.05953	23.199642	7.296702	2.192713	13.691866	1.4391	0.01971
Ctr9	ENSMUSG00000005609	27.277414	16.903906	18.601816	3.369417	4.855728	0.961501	2.77277	0.00632
Slc25a12	ENSMUSG00000027010	21.39879	24.139307	16.716934	7.904333	4.529452	9.726735	1.4902	0.00725
Garnl3	ENSMUSG00000038860	23.540159	20.51544	17.905348	5.498259	0.645677	0.531263	3.21448	0.00134
Cir1	ENSMUSG00000041777	13.901576	21.022522	26.741934	12.428931	0	3.247894	1.97584	0.04343
Asic2	ENSMUSG00000020704	19.105059	19.818668	21.11203	6.966971	10.218257	7.838257	1.26254	0.00051
Dpp7	ENSMUSG00000026958	16.994438	26.574514	15.939889	11.234605	0.193424	0	2.38053	0.03319
Ctcf	ENSMUSG00000005698	17.418032	21.612976	20.410181	11.727396	2.298717	9.087529	1.36272	0.01695
Dnajc9	ENSMUSG00000021811	18.613619	20.812967	19.973589	14.495327	8.167205	2.727674	1.2262	0.0306
130071C03Rik	ENSMUSG00000050334	21.42787	22.808577	15.016132	4.199716	1.557489	0	3.36344	0.00271
Ccser2	ENSMUSG00000058690	24.403786	16.161055	18.371506	9.407201	1.286544	7.53102	1.69326	0.01751
Paqr7	ENSMUSG00000037348	21.33308	20.955559	16.245216	2.097322	2.074687	2.353009	3.16522	0.00045
Ppie	ENSMUSG00000028651	12.777855	18.655903	26.864525	0	0.356117	4.555842	3.56908	0.01484
Fars2	ENSMUSG00000021420	23.404308	21.956221	12.815603	9.819453	0	3.403157	2.13742	0.02695
Cpne8	ENSMUSG00000052560	26.603415	18.230078	13.214912	6.857434	7.192349	0	2.04671	0.03228
Thoc3	ENSMUSG00000025872	20.522903	19.972748	17.233238	3.212293	0	10.965026	2.02571	0.01308
Rxrb	ENSMUSG00000039656	28.642263	17.381359	11.658653	1.156874	2.611798	2.597241	3.17969	0.02696
Wdr73	ENSMUSG00000025722	14.6586	21.86624	20.982306	2.720119	0	10.324162	2.14032	0.01805
Gm10419	ENSMUSG00000072769	16.382139	21.250731	19.821442	8.826634	2.250282	9.347555	1.49212	0.01028
Lin52	ENSMUSG00000085793	22.95619	22.017754	12.25684	4.742258	8.754632	0.308336	2.05158	0.02617
Ano10	ENSMUSG00000037949	24.855209	14.418308	16.606155	6.021456	0.024283	4.889542	2.35333	0.0151
Rtf2	ENSMUSG00000027502	14.640194	20.14122	20.826061	2.213939	0.31277	12.413323	1.8961	0.03291
Me3	ENSMUSG00000030621	10.328153	26.052567	18.401508	2.290636	0.492025	8.059343	2.33708	0.0449
Cnn3	ENSMUSG00000053931	15.491528	21.906858	15.844261	5.020833	6.275487	0.032988	2.23252	0.00777
Zfp131	ENSMUSG00000094870	16.088312	18.808268	18.157139	11.072066	0.582865	8.911657	1.36715	0.03049
Utp4	ENSMUSG00000041438	11.011786	21.655128	18.919123	2.591415	4.054442	5.797172	2.05164	0.01715
Eif3j1	ENSMUSG00000027236	18.878887	20.302235	12.02739	11.167554	3.413477	6.202949	1.30091	0.04118
Gm5620	ENSMUSG00000056904	12.369367	18.084885	20.383841	7.989491	3.353618	10.500121	1.21872	0.03812
Rhot1	ENSMUSG00000017686	16.313522	22.210588	12.238066	0	9.878424	1.533109	2.15326	0.03591
Lor	ENSMUSG00000043165	25.833115	18.257431	6.658538	0	0	2.049155	4.63028	0.04457
Slc2a8	ENSMUSG00000026791	18.559063	13.004018	18.710752	5.632187	2.703175	0	2.59249	0.0049

Snhg4	ENSMUSG00000117869	15.209075	7.968777	26.809639	2.853141	0	0.070551	4.0957	0.04793
Eipr1	ENSMUSG00000036613	9.771556	19.075832	21.103073	7.061944	5.826927	2.704167	1.6796	0.03704
Ak3	ENSMUSG00000024782	25.790274	9.426388	13.872313	0.620462	4.319375	0.795598	3.09742	0.04537
Slc45a1	ENSMUSG00000039838	15.186409	16.154974	17.338364	0	5.132829	0.864749	3.02087	0.00113
Fam3a	ENSMUSG00000031399	14.948997	20.002081	13.244294	0.012593	0	10.884891	2.1449	0.04026
Ppm1h	ENSMUSG00000034613	13.484984	11.809865	22.889719	6.673489	0.115584	5.640697	1.95477	0.04089
Rab8a	ENSMUSG00000003037	22.499107	13.631007	11.939391	3.300237	0	3.927797	2.73345	0.01759
Arhgef7	ENSMUSG00000031511	14.455695	16.061447	17.538807	3.105162	2.524973	0.105686	3.06664	0.00038
Fam120b	ENSMUSG00000014763	13.612619	16.867884	17.503807	12.077289	6.623906	4.513803	1.04751	0.03195
Ercc1	ENSMUSG00000003549	18.977051	11.685495	16.463806	2.325573	2.927909	0	3.16519	0.00383
Gm47441	ENSMUSG00000113606	15.286918	17.343925	14.422771	4.267965	4.854749	8.738603	1.39747	0.00412
Eme2	ENSMUSG00000073436	18.669748	15.951401	12.292931	1.456016	0.738258	6.26746	2.471	0.0072
Mau2	ENSMUSG00000031858	16.955969	18.472372	11.145333	5.449067	1.044705	2.20479	2.42067	0.00823
Ecd	ENSMUSG00000021810	11.361831	17.41674	17.451262	0.397051	5.90545	8.71053	1.62261	0.03048
Rhof	ENSMUSG00000029449	7.407744	17.366467	20.948944	1.810906	0.142827	2.011545	3.52743	0.02728
Thoc5	ENSMUSG00000034274	16.132095	17.270123	12.215802	9.316205	0.02629	4.577435	1.71245	0.02674
Rnf170	ENSMUSG00000013878	20.637592	7.080869	16.392868	5.903029	0	0.571665	2.76827	0.04703
Pomk	ENSMUSG00000037251	13.913059	13.524935	16.501551	1.314615	0	0	5.06281	0.00016
Fbxo34	ENSMUSG00000037536	13.164085	12.736329	17.831116	0.012948	0.021139	0	10.3252	0.00087
Sgcb	ENSMUSG00000029156	15.665111	15.968971	11.66474	0.596344	3.206976	4.845355	2.32378	0.00341
Kpna1	ENSMUSG00000022905	11.997189	14.598155	16.638063	8.60281	1.928905	4.902369	1.48603	0.01695
Gm4828	ENSMUSG00000116620	17.525269	12.419788	12.613726	7.774629	5.29832	6.905581	1.09101	0.01446
Setd5	ENSMUSG00000034269	14.540072	10.460504	17.214727	5.763554	2.705235	8.169648	1.34325	0.02775
Noct	ENSMUSG00000023087	12.183134	13.74813	15.977148	3.453214	1.626718	1.396117	2.69405	0.00076
Slc30a5	ENSMUSG00000021629	8.342776	18.759451	14.741647	0.712947	1.096942	3.013536	3.11689	0.01666
Ing1	ENSMUSG00000045969	15.919475	10.682957	15.149872	9.23712	0	1.213196	1.99831	0.03495
Dusp12	ENSMUSG00000026659	8.513997	20.500938	12.688569	4.899232	0	1.349968	2.73843	0.03601
Plekha7	ENSMUSG00000045659	19.968201	10.629793	10.973904	6.949164	0	1.335017	2.32718	0.0408
Lpl	ENSMUSG00000015568	10.593526	19.090584	10.446502	0.004814	0.120588	0.004498	8.27116	0.00954
Apba3	ENSMUSG00000004931	16.803692	9.970682	13.153345	0.665663	2.791813	0.760897	3.24263	0.00471
Snx12	ENSMUSG00000046032	15.454636	15.247361	8.651024	7.757799	0	3.458681	1.81085	0.04147
Slc25a44	ENSMUSG00000050144	8.036668	18.651358	12.125349	0.18488	0	5.942579	2.66319	0.04071
Crppa	ENSMUSG00000043153	13.562392	6.46714	18.654049	1.610505	0	4.808744	2.59124	0.04755
Cpox	ENSMUSG00000022742	13.549317	13.400527	11.540494	2.705711	0	6.299713	2.09563	0.00709
Slc1a4	ENSMUSG00000020142	12.157848	14.786344	11.480447	9.228416	1.616402	1.815998	1.60166	0.03351
Ctc1	ENSMUSG00000020898	13.953049	16.128	7.706631	1.36443	0	0.950292	4.02901	0.00984
Cadm3	ENSMUSG00000005338	12.80322	13.269804	11.499301	8.15886	3.132304	3.956558	1.30108	0.01061
Zfp451	ENSMUSG00000042197	13.210711	9.341152	14.963879	7.214811	5.746511	5.734276	1.0048	0.02233
Nup54	ENSMUSG00000034826	12.250813	10.407381	14.77995	6.366295	0	0.473948	2.45239	0.01331
Nos1	ENSMUSG00000038473	12.204362	17.081469	8.082341	1.543452	0	0.146867	4.46644	0.01088
Abcb8	ENSMUSG00000028973	10.382494	11.757296	13.502681	5.049438	0	1.015852	2.55495	0.00527
Slc19a2	ENSMUSG00000040918	14.26732	15.051285	5.778086	0	1.890079	0	4.21482	0.02183
Ptbp1	ENSMUSG00000006498	9.676013	16.291327	8.898912	0.542691	0.126085	0	5.70416	0.00835
Gm35612	ENSMUSG00000119984	18.296392	7.962934	8.577496	0	0	0.028413	10.2598	0.02566
Mcu	ENSMUSG00000009647	13.144123	10.08945	11.602135	3.965438	0	8.557485	1.476	0.04719
Gm13461	ENSMUSG00000081999	14.286654	8.74841	11.70357	6.307454	4.16285	6.61615	1.02369	0.02958
Gfra2	ENSMUSG00000022103	12.595716	10.287539	11.833529	6.046809	0	1.401748	2.2206	0.00957
Mok	ENSMUSG00000056458	16.871412	9.954693	7.667479	0	2.773067	0	3.63677	0.02226
Otulin	ENSMUSG00000046034	11.331159	15.166232	7.577648	0	0	0.829119	5.36099	0.00739
Fbll1	ENSMUSG00000051062	13.948698	11.247448	8.330076	5.838289	0.067764	0.035199	2.49645	0.02181
Prmt5	ENSMUSG00000023110	11.107961	13.060439	9.313651	4.643202	0	4.385458	1.8908	0.01174
Ankle2	ENSMUSG00000029501	11.488299	9.540135	12.22923	4.853893	3.507149	1.113791	1.81151	0.00427
Nanos1	ENSMUSG00000072437	13.195759	13.669587	6.202769	1.096838	5.83121	2.67121	1.78445	0.04844
Atg4d	ENSMUSG00000002820	17.363079	10.790236	4.706144	0.020683	0.154361	0.757215	5.13943	0.04383
Gm57050	ENSMUSG00000121068	9.395365	6.384292	16.991423	1.367681	1.188077	2.876003	2.59293	0.04654
Gm37728	ENSMUSG00000103220	11.229896	8.65641	12.355848	3.14176	8.199456	4.001347	1.07141	0.04186
Csmd2	ENSMUSG00000028804	8.113982	13.272686	10.551925	6.229783	0.2747	2.389453	1.84441	0.02857
Myrip	ENSMUSG00000041794	11.309257	11.797857	8.341063	0	0.125158	0	7.97308	0.00064
Ss18l1	ENSMUSG00000039086	10.009982	14.519774	6.854875	3.38204	0	3.353073	2.22028	0.02999
Fscn1	ENSMUSG00000029581	15.986688	7.847397	7.532673	2.262447	2.216464	0	2.80802	0.03528
Reno1	ENSMUSG00000075389	13.279485	5.172485	12.661999	0	0.643848	0	5.5947	0.01777
Gm9234	ENSMUSG00000058050	10.736325	8.666958	11.684646	5.342147	3.488881	5.393067	1.12802	0.00669
Sms-ps	ENSMUSG00000081752	7.452034	11.204758	11.347141	3.030971	6.204281	2.769935	1.32149	0.02365
